# Bio-optimized *Curcuma longa* extract is efficient on knee osteoarthritis pain: a double-blind multicenter randomized placebo controlled three-arm study

**DOI:** 10.1186/s13075-019-1960-5

**Published:** 2019-07-27

**Authors:** Y. Henrotin, M. Malaise, R. Wittoek, K. de Vlam, J.-P. Brasseur, F. P. Luyten, Q. Jiangang, M. Van den Berghe, R. Uhoda, J. Bentin, T. De Vroey, L. Erpicum, A. F. Donneau, Y. Dierckxsens

**Affiliations:** 1 Bone and Cartilage Research Unit, Arthropôle Liège, Institute of Pathology, Level 5, CHU Sart-Tilman, University of Liège, 4000 Liège, Belgium; 2 Department of Physical Therapy and Rehabilitation, Princess Paola Hospital, Vivalia, Marche-en-Famenne, Belgium; 3 Artialis SA, GIGA Tower, CHU-Sart-Tilman, 4000 Liège, Belgium; 4 Rheumatology Department, CHU Sart-Tilman, Liège, Belgium; 5 Rheumatology Department, UZ Gent, Ghent, Belgium; 6 Rheumatology Department, ZNA Jan Palfijn, Merksem, Belgium; 7 Rheumatology Department, CHU UCL Namur, Yvoir, Belgium; 8 Rheumatology Department, University Hospitals Leuven, Leuven, Belgium; 9 Rheumatology and Physical Medicine Department, Hôpitaux Iris Sud, Bruxelles, Belgium; 10 Rheumatology Department, Algemeen Stedelijk Ziekenhuis, Aalst, Belgium; 11 Physical Medicine and Rehabilitation Department, Centre Hospitalier du Bois de l’Abbaye, Seraing, Belgium; 12 Rheumatology Department, CHU Brugmann, Bruxelles, Belgium; 13 Physical Medicine, UZA, Antwerpen, Belgium; 14 Anthisnes, Belgium; 15 Public health Science Department, University of Liège, Liège, Belgium; 16 Tilman SA, Baillonville, Belgium

**Keywords:** Osteoarthritis, Biomarkers, Curcumin

## Abstract

**Objectives:**

Comparison of two doses of bio-optimized *Curcuma longa* extract (BCL) in the management of symptomatic knee osteoarthritis (OA).

**Methods:**

A prospective, randomized, 3-month, double-blind, multicenter, three-group, placebo-controlled trial assessing Patient Global Assessment of Disease Activity (PGADA) and serum sColl2-1, a biomarker of cartilage degradation, as co-primary endpoints. Pain on visual analog scale (VAS), Knee injury and Osteoarthritis Outcome Score (KOOS), and paracetamol/non-steroidal anti-inflammatory drug (NSAID) consumption were used as secondary endpoints.

**Results:**

One hundred fifty patients with knee OA were followed for 90 days. Low and high doses of BCL showed a greater decrease of PGADA than placebo. Analysis of sColl2-1 showed in the placebo and BCL low-dose groups, but not in the BCL high-dose group, a transient but non-significant increase of sColl2-1 between T0 and T1. Thereafter, in all groups, sColl2-1 decreased between T1 and T3 (all *p* < 0.01), but no difference between the groups was found. Pain reduction at day 90 in the low- and high-dose BCL groups (− 29.5 mm and − 36.5 mm) was higher than that in the placebo (− 8 mm; *p* = 0.018). The global KOOS significantly decreased overtime, but changes were comparable across treatment arms. The ratio of patients with adverse events (AE) related to the product was similar in the placebo and treatment groups, but the number of AE linked to the product was higher in the high-dose BCL group compared to the placebo (*p* = 0.012).

**Conclusions:**

BCL appeared safe and well-tolerated with no evidence of severe adverse effects. Efficacy analysis suggested positive trends for measurements of PGADA and serum levels of an OA biomarker and showed a rapid and significant decrease of pain in knee OA (Trial registration: ISRCTN, ISRCTN12345678. Registered 21 September 2016—retrospectively registered, https://clinicaltrials.gov/ct2/show/NCT02909621?term=osteoarthritis+curcumin&rank=5—Evaluation of FLEXOFYTOL® Versus PLACEBO (COPRA) NCT02909621).

**Electronic supplementary material:**

The online version of this article (10.1186/s13075-019-1960-5) contains supplementary material, which is available to authorized users.

## Key messages

- This is the first RCT respecting the clinical guidance investigating the effects of bio-optimized *Curcuma longa* extract in knee OA patients.
- Bio-optimized *Curcuma longa* extract is an efficient and safe treatment to relieve pain in knee OA patients.
- Bio-optimized *Curcuma* significantly decreases paracetamol and non-steroidal anti-inflammatory drug consumption.

## Introduction

Osteoarthritis (OA) affects 240 million people globally, about 10% of men and 18% of women [1]. Clinically, OA is characterized by pain, transient morning stiffness, and crepitus with joint motion, all of which deteriorate daily quality of life, leading to sedentary and increased morbidity and mortality [2, 3]. The prevalence of OA is rising due to the aging population and growing rates of obesity [4]. Up to date, there is no curative treatment for OA despite the availability of a large number of therapeutic options, including non-pharmacological, pharmacological, and surgical therapies. Recommendations for the management of knee OA have been published by numerous scientific and medical societies [5–8]. Briefly, the association of non-pharmacological and pharmacological modalities for optimal management of OA is recommended. The pharmacological treatment of OA is mostly symptomatic to relieve pain and improve function. The first-line pharmacological therapy is the use of paracetamol and non-steroidal anti-inflammatory drugs (NSAIDs). However, these drugs have a low transient analgesic effect on knee OA, and some meta-analysis indicated the occurrence of adverse events mainly in elderly patients with co-morbidities [9–12]. In particular, safety profiles of oral NSAIDs remain a concern, and caution is recommended before selecting an NSAID and dose regimen for a patient [13]. For that reason, it is recommended to use NSAIDs at the lowest efficient dose and intermittently [8, 14]. The unfavorable safety profiles of commonly prescribed knee OA treatments have led patients, clinicians, and industry to seek safer alternatives including dietary supplements like curcumin [15, 16]. Curcumin (diferuloylmethane) is the principal curcuminoid extracted from the *Curcuma longa* (turmeric) root [17]. Curcumin has been identified as an inhibitor of the nuclear factor-kappa β (NF-κβ) pathway and a scavenger of reactive oxygen and nitrogen species [18, 19]. Two recent meta-analyses evaluating a large number of dietary supplements ranked curcumin as one of the most effective compounds for pain reduction in OA at short term although the quality of evidence was very low [20, 21]. Curcuminoids showed no statistically significant differences in the efficacy outcomes compared to NSAIDs, but patients receiving curcuminoids were significantly less likely to experience gastrointestinal adverse events [21]. A meta-analysis of different turmeric derivatives including curcumin [22] reported the positive influence of bio-optimization by adjuvants such as piperine, liposomal delivery systems, phospholipid complexes of curcumin, or use of polysorbate as an emulsifier, which is relevant since curcumin’s low bioavailability is regarded as a major challenge for its optimal effectiveness.

Herein, we report results from a study of pharmaceutical-grade bio-optimized *Curcuma longa* extract in patients with symptomatic knee OA, which, to our knowledge, is the first ever to have been conducted in full accordance with the International Council for Harmonisation of Technical Requirements for Pharmaceuticals for Human use (ICH E6).

## Population and methods

### Study design and selection of patients

This study included patients from Belgium enrolled by 11 physicians between September 2014 and June 2017 and was conducted by an independent contract research organization (Artialis SA, Liège, Belgium). The main inclusion criteria were age between 45 and 80 years with primary femorotibial and/or femoropatellar symptomatic knee OA diagnosed according to the clinical and radiological criteria of American College of Rheumatology (ACR) [23]. The more symptomatic knee (with a pain score of at least 40 mm on a 0–100-mm visual analog scale (VAS) for at least 3 months before enrollment) was defined as the target knee. The main exclusion criteria were dementia or inability to give informed consent, pregnancy or lactation, planned knee replacement surgery, allergy or contraindication to curcumin, recent trauma of the knee responsible of the symptomatic knee, and joint disease other than OA. Use of any intra-articular injection in the target knee in the last 3 months, symptomatic slow-acting drugs in OA (SYSADOA) in the last month, oral corticotherapy ≥ 5 mg/day in the last 3 months, products with curcuminoids extract in the last 3 months, anticoagulant (coumarinic compound), and heparin (both relative contraindication for BCL) was also specifically forbidden by the study protocol. Patients had to be regular users of paracetamol and/or oral NSAIDs to manage OA knee pain and were authorized to continue the intake of these drugs during the study. Ethics Committee approval from all participating centers was obtained, and all patients gave their written informed consent to participate.

### Treatment assignment

Patients were randomly assigned to one of the following three groups with a ratio of 1:1:1: (1) placebo 2 × 3 caps/day, (2) bio-optimized *Curcuma longa* extracts (BCL) low dosage 2 × 2 caps/day plus placebo 2 × 1 cap/day, and (3) BCL high dosage 2 × 3 caps/day. Capsules were taken twice daily with water, once at breakfast and once at dinner for 3 months. This pharmaceutical-grade BCL has received approval from Belgian Competent Authorities (PL 31/100; Federal Public Service, Health, Food Chain Safety and Environment) and commercialized under the brand name FLEXOFYTOL® (Tilman SA, Baillonville, Belgium). The capsule of BCL available on the market was encapsulated to allow for a double-blind design. The allocation sequence was created by restricted blocking randomization, with a block size of 6. To achieve a good balance between the treatments delivered to each center, investigation kits were shipped by blocks, which were multiple of 3. The investigator was asked to distribute the blinded kit by following the randomization sequence, in order to achieve a good balance between the three treatment groups within this center. Each capsule contained 46.67 mg of turmeric rhizome extract (*Curcuma longa* L.), Polysorbate 80 [E433] as an emulsifier, and citric acid [E330] as an acidity regulator. Placebo capsules contained sunflower seed oil. For rescue analgesia, patients were allowed to take paracetamol 500 mg tablets (maximum dosage 3 g/day) or oral NSAIDs if paracetamol was insufficient. An appropriate washout period of 24 h was required before symptom assessment at in-clinic visits. No other pharmacological or non-pharmacological interventions for OA were allowed. Compliance with the study treatments was established by counting unused study products. Usual treatments taken by the patient in other non-OA-related diseases were allowed; anticoagulants (coumarinic compounds) and heparin were forbidden.

### Outcome measures

There were two co-primary endpoints, and both were assessed as the change from baseline, that is, the difference between the enrollment and study conclusion. One endpoint was the Patient Global Assessment of Disease Activity (PGADA) on a 100-mm visual analog scale (VAS). The other endpoint was the serum levels of Coll2-1 (sColl2-1), a specific amino acid sequence located in the triple helicoidal part of type II collagen and considered as a biomarker of cartilage degradation [24, 25]. sColl2-1 was measured in diluted serum using an enzyme-linked immuno-sensitive assay ELISA method (Artialis SA, Liège, Belgium). The secondary endpoints included the patient’s estimate of pain on a 100-mm VAS, the Knee injury and Osteoarthritis Outcome Score (KOOS) index, and its subscale scores using a self-administered questionnaire. Paracetamol/NSAID consumption during the month prior to each visit and patients’ satisfaction with the treatment using a 5-point ordinal Likert scale (better, little better, none, little lower, or far lower). All adverse events (AE) and abnormal laboratory test results were recorded.

### Statistical analysis

Statistical analyses have been carried out following the recommendations and guidance on statistical principles for clinical trials (Sakpal, 2010; ICH E9) (Additional file 2). The sample size was considering the results obtained in a previous pilot study [26]. We calculated a sample size of 50 patients per group (150 patients in total) based on a difference in Coll2-1 biomarker level of 44.4 nmol/l with a standard deviation of 53 and a difference in patient assessment of global disease activity of 21 mm with a standard deviation of 25, an alpha risk of 2.5% (to take into account multiplicity due to co-primary endpoints), a power of 85%, and a dropout rate of 15%. Mixed model (repeated measures ANCOVA model) followed by post hoc adjustments test was used to evaluate the efficacy of primary and secondary target variables. *p* values for treatment effect, time effect, and the effect of their interaction were considered significant at the 2.5% alpha level for co-primary endpoints (*p* < 0.025) and at the 5% alpha level for the secondary endpoints (*p* < 0.05).

As no difference between the BCL treated groups was seen at baseline and at 3 months and as curcumin levels in the serum were not significantly different between BCL doses, pooled values of the BCL-treated groups were also considered. PGADA and sColl2-1 from baseline to month 3 were compared between the three treatment groups by means of a linear mixed model. In some situations, a log transformation has been considered for achieving normal distribution of the outcome of interest. Within the longitudinal framework, between-group differences have been investigated at each time point using classical post hoc test. Differences in the distribution of qualitative criteria between the treatment groups have been analyzed using the chi-square (*χ*^2^) test. Compliance has been evaluated by the pill count method, as well as in percentage of the estimated versus theoretical consumption. Post hoc analysis included the longitudinal analyses of sColl2-1, PGADA, VAS pain, and KOOS using raw data and absolute difference; the longitudinal analyses of Coll2-1, PGADA, and patient’ estimated pain using pooled values of the BCL-treated groups; and the longitudinal analyses of PGADA and VAS pain using adjusted value for the intake of rescue treatment. All results were considered to be significant at the 5% critical level (*p* < 0.05). Statistical analyses were carried out using the Statistical Analysis System (SAS version 9.4, SAS Institute Inc., Cary, NC, USA). The following analysis populations were defined for the study: the safety population (SP) includes all patients treated with at least one dose of the investigational product. The safety analysis was based on the safety population. Intention to treat (ITT) population includes every subject who is randomized according to randomized treatment assignment. Full analysis set (FAS) population comprises of subjects who have one efficacy basal measure and at least one corresponding post-baseline efficacy measurement for one of the main efficacy variables, regardless of subsequent withdrawal from treatment or deviation from the protocol. Per-protocol (PP) population included all treated subjects who had not suffered any major protocol deviation.

## Results

**Fig. 1:**
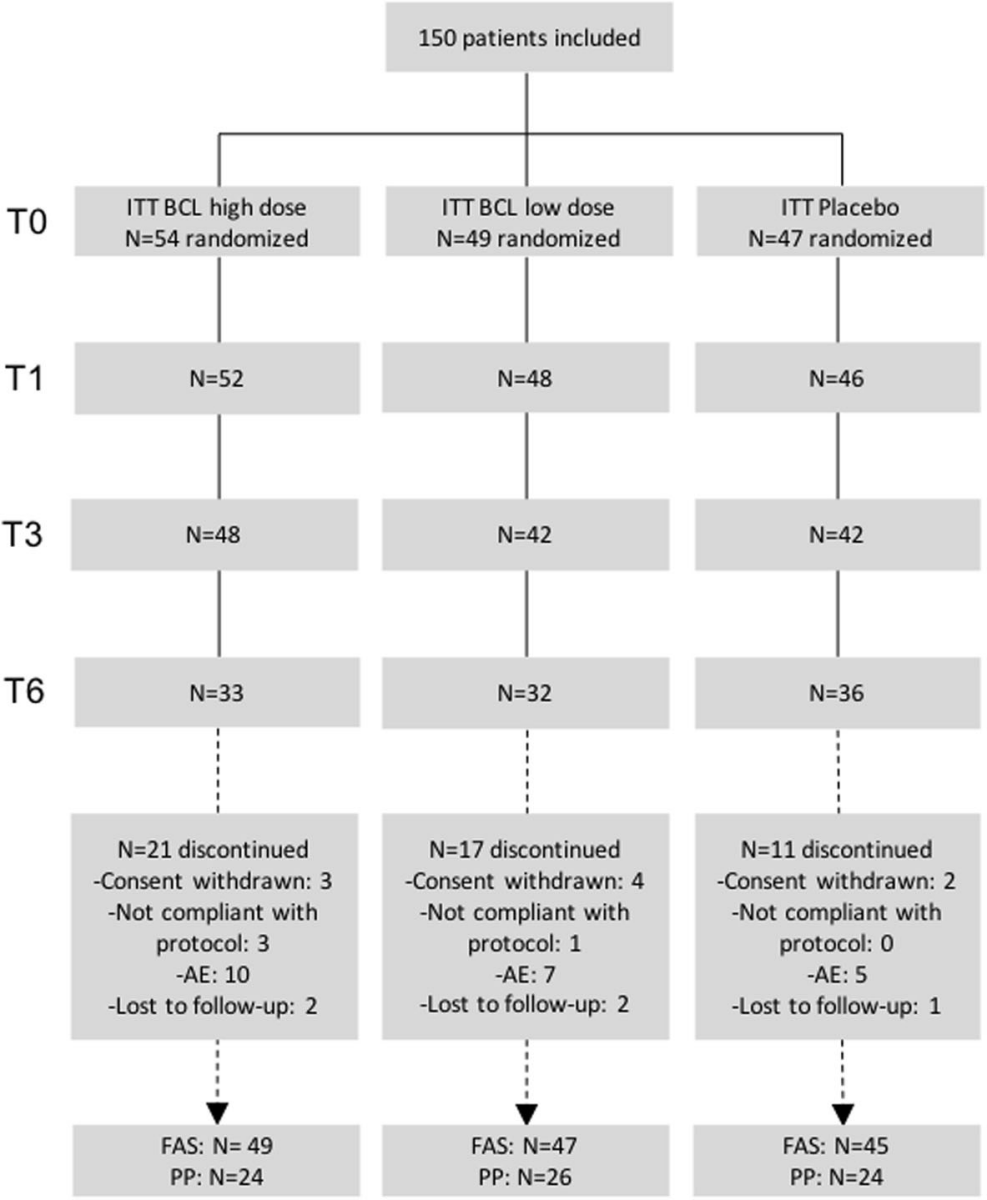
Disposition of patients. BCL, bio-optimized *Curcuma longa*; FAS, full analysis set; ITT, intention to treat; PP, per-protocol; AE, adverse event

One hundred fifty patients were randomized and considered eligible for the ITT analysis and 141 for the FAS analysis. Among the FAS population, 49 received a high dose of BCL, 47 received a low dose of BCL, and 45 received placebo (Fig. 1). The cumulative time distribution of withdrawals was similar in the three groups without significant differences in the reasons for withdrawals. At baseline, participants in each group were well-matched (Table 1). Grades II to IV of KL were equally distributed between the three groups, with 99% of grade II and III. At inclusion, 92.2% of patients were taking paracetamol and 71.6% NSAIDs. All the patients were using paracetamol and/or NSAIDs during the study period. The majority (82.3%) of paracetamol and NSAID consumers were taking these drugs between 7 to 14 days a month.

**Table 1:** Demographic and baseline characteristics of patients

	BCL high dose, *N* = 49	BCL low dose, *N* = 47	Placebo, *N* = 45
Age (years)			
Mean (SD)	60.9 (9.78)	61.4 (7.49)	63.3 (7.69)
Sex, *n* (%)			
Female	39 (79.6)	40 (85.1)	34 (75.6)
BMI (kg/m^2^)			
Mean (SD)	29.4 (4.87)	30.4 (5.23)	29.4 (5.2)
Time from diagnosis of knee OA (years)			
Mean (SD)	7.41 (7.294)	6.6 (4.671)	7.6 (9.3)
KL grade, *n* (%)			
Grade I	0 (0.0%)	0 (0.0%)	0 (0.0%)
Grade II	31 (63.3%)	27 (57.4%)	29 (64.4%)
Grade III	18 (36.7%)	19 (40.4%)	16 (35.6%)
Grade IV	0 (0.0%)	1 (2.0%)	0 (0.0%)
Target knee (the most symptomatic), *n* (%)			
Right	27 (55.1%)	22 (46.8%)	25 (55.6%)
Target knee pain (VAS, mm)			
Mean (SD)	62.9 (13.8)	63.3 (15.8)	59.9 (12.3)
PGADA (VAS, mm)			
Mean (SD)	67.2 (17.5)	69.6 (17.8)	63.9 (17.3)
Coll2-1 (nM)			
Mean (SD)	393.39 (165.2)	373.8 (133.5)	384.7 (122.0)
Ultra-sensitive CRP (mg/l)			
Mean (SD)	2.5 (2.5)	3.4 (2.9)	3.2 (2.8)
Rescue treatment (NSAIDs) in the last month, *n* (%)			
No	15 (30.6%)	12 (25.5%)	13 (28.9%)
Less than 7 days	5 (10.2%)	2 (4.3%)	2 (4.4%)
7 to 14 days	15 (30.6%)	19 (40.4%)	16 (35.6%)
15 to 21 days	4 (8.2%)	9 (19.1%)	5 (11.1%)
More than 21 days	7 (14.3%)	2 (4.3%)	6 (13.3%)
Every day	2 (4.1%)	3 (6.4%)	3 (6.7%)
NI	1 (2.0%)	0 (0.0%)	0 (0.0%)
Rescue treatment (paracetamol) in the last month, *n* (%)			
No	6 (12.2%)	3 (6.4%)	2 (4.4%)
Less than 7 days	1 (2.0%)	3 (6.4%)	2 (4.4%)
7 to 14 days	27 (55.1%)	18 (38.3%)	21 (46.7%)
15 to 21 days	3 (6.1%)	11 (23.4%)	8 (17.8%)
More than 21 days	4 (8.2%)	6 (12.8%)	7 (15.6%)
Every day	8 (16.3%)	6 (12.8%)	5 (11.1%)
NI	6 (12.2%)	3 (6.4%)	2 (4.4%)
KOOS—global score			
Mean (SD)	199.2 (68.1)	201.5 (73.5)	197.7 (60.0)
KOOS—pain			
Mean (SD)	45.3 (16.4)	45.8 (15.6)	44.2 (13.9)
KOOS—ADL			
Mean (SD)	49.8 (17.9)	48.9 (17.3)	48.9 (16.6)
KOOS—QoL			
Mean (SD)	32.0 (18.7)	31.5 (19.2)	33.3 (18.1)
KOOS—symptoms			
Mean (SD)	52.15 (15.9)	52.8 (15.6)	54.1 (13.5)
KOOS—Sport and rec.			
Mean (SD)	19.5 (17.1)	19.8 (19.8)	17.2 (14.5)

*ADL* activity of daily life, *BMI* Body Mass Index, *NSAID* non-steroidal anti-inflammatory drug, *KL* Kellgren-Lawrence, *NI* no information, *OA* osteoarthritis, *PAGDA* Patient Global Assessment of Disease Activity, *QoL* Quality of life, *SD* Standard Deviation, *VAS* Visual Analog Scale

ANCOVA model for repeated measures was performed to analyze the two co-primary endpoints as well as the secondary endpoints on FAS and PP populations. Longitudinal differences such as pain relief and improvement of PGADA were detected for all groups, but no significant difference between the treatment was highlighted. Results for FAS population were shown in Additional file 1: Table S1. Nevertheless, post hoc tests revealed interesting results and were presented here after.

**Fig. 2:**
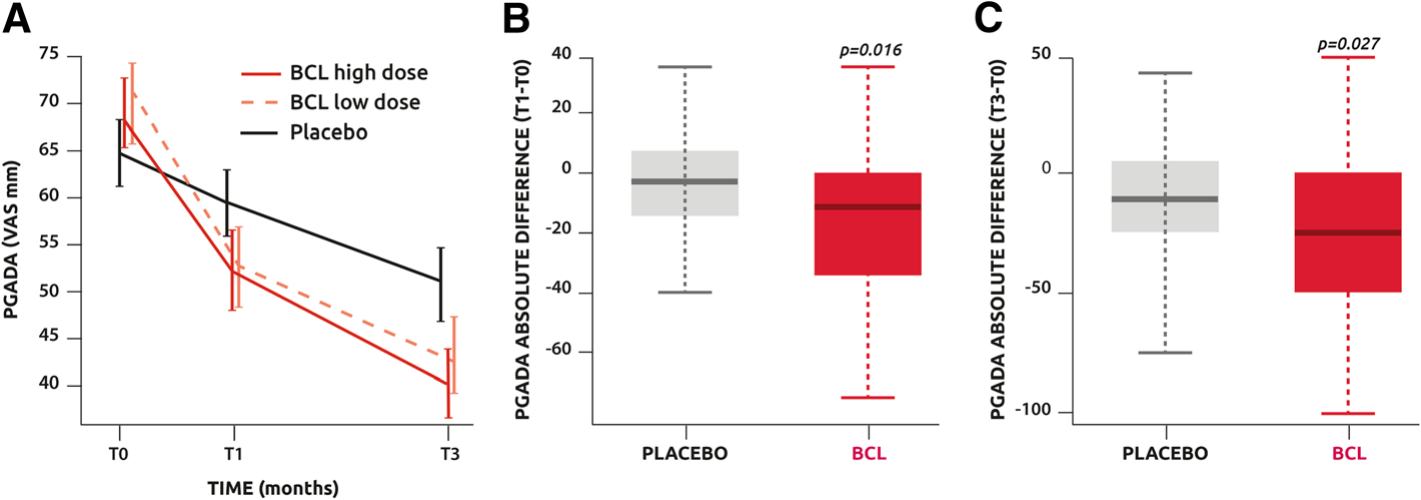
**a** Patients Global Assessment of Disease Activity (PGADA) assessed with a VAS evolution with time. **b** Comparison of the absolute difference of PGADA between T0 and T1 in the placebo and pooled low- and high-dose BCL groups. **c** Comparison of the absolute difference of PGDA between T0 and T3 in the placebo and pooled low- and high-dose BCL groups

Analysis of PGADA on the ITT population revealed a significant improvement in all treatment arms compared with time (all groups *p* < 0.0001), but the time evolution curves were found comparable (Fig. 2a). Analysis of absolute difference between baseline and time points on the pooled treated patients in FAS revealed a significantly greater reduction of PGADA in treated patients than in the placebo group at T1 and T3 (*p* = 0.016 and *p* = 0.027, respectively) (Fig. 2b, c).

**Fig. 3:**
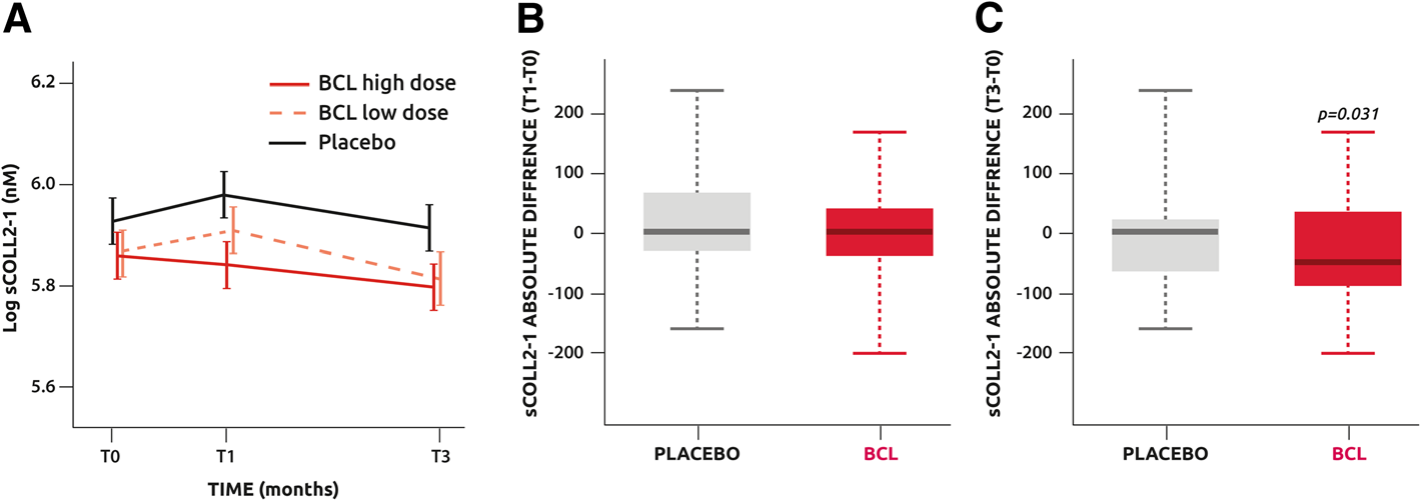
**a** Serum levels of Coll2-1 evolution with time. **b** Comparison of the absolute difference of sColl2-1 between T0 and T1 in the placebo and pooled low- and high-dose BCL groups. **c** Comparison of the absolute difference of sCOll2-1 between T0 and T1 in the placebo and pooled low- and high-dose BCL groups

Analysis of sColl2-1 on the ITT population showed in the placebo and BCL low-dose groups, but not in the BCL high-dose group, a transient but non-significant increase of sColl2-1 between T0 and T1. Thereafter, in all groups, sColl2-1 decreased between T1 and T3 (all *p* < 0.01), but no difference between the groups was found (Fig. 3a). Of note, the baseline level of sColl2-1 was similar between the groups. Using pooled values for the treated groups (BCL high plus low doses) on PP population, an absolute difference of sColl2-1 between baseline and T3 revealed a significant difference between the pooled BCL groups and placebo (*P* = 0.031) (Fig. 3b, c).

**Fig. 4:**
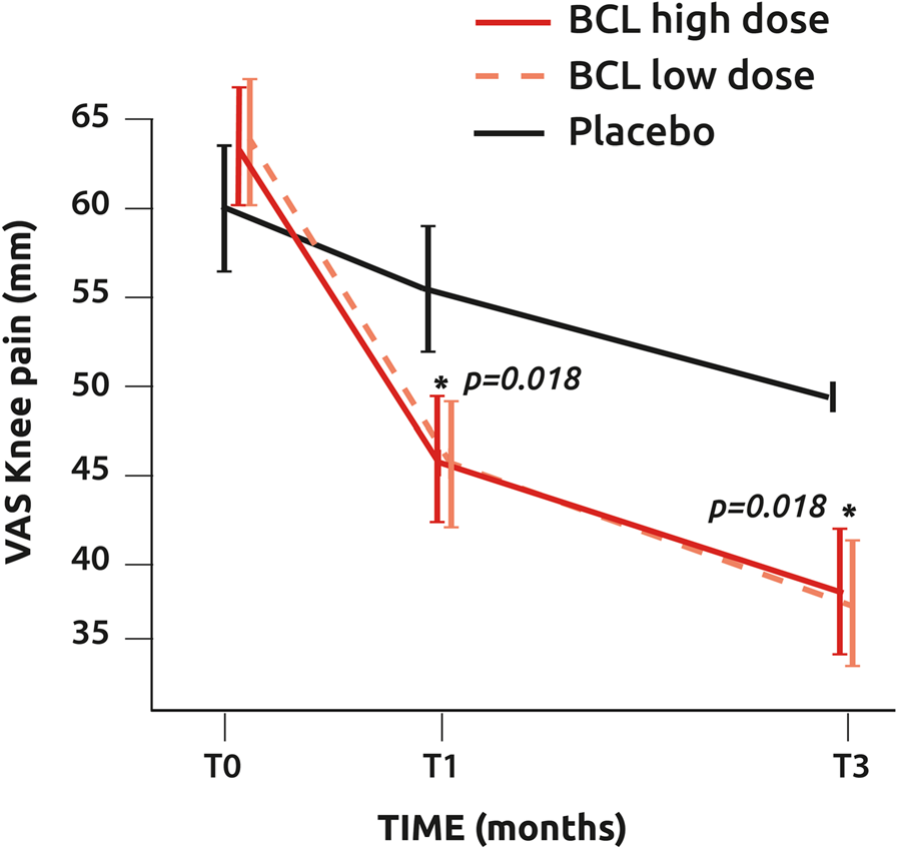
Knee pain assessment with a VAS evolution with time

In ITT, knee pain on VAS significantly decreased with time (*p* < 0.001) in both BCL high and low doses (Fig. 4) while significance was not achieved after 3 months in the placebo group (*p* = 0.051). At T3, a significant difference was observed in ITT between both BCL high dose and low dose and placebo (both *p* = 0.018), while no significant difference was found between both BCL doses (*p* = 0.9002). The absolute difference between baseline and time points was significantly more important in the BCL groups than in placebo at T1 (high dose − 16 mm, low dose − 16.5 mm, placebo − 4 mm; *p* = 0.046) and T3 (high dose − 29.5 mm, low dose − 36.5 mm, placebo − 8 mm; *p* = 0.032). The improvement in pain observed in the BCL low-dose group corresponds to an effect size (ES) of 0.35 for pain at T1 and 0.43 at T3.

KOOS global score and subscales were significantly improved over time in all groups (*p* < 0.001), and the evolution was found comparable within the three groups in the FAS population (Table 2). Although KOOS global score and subscale changes to T1 and T3 tended to be more important in the BCL groups, no significant difference between the groups was found.

**Table 2:** KOOS changes between the follow-up visits (T3 and T1) and baseline FAS population

		BCL high dose, mean ± SD	BCL low dose, mean ± SD	Placebo, mean ± SD
KOOS change at T1 (difference between T1 and baseline)	*N*	49	46	45
	Global score	35.2 ± 67.5	18.0 ± 57.6	7.9 ± 60.2
	Pain	7.1 ± 17.5	4.8 ± 16.7	3.1 ± 13.9
	Symptoms	6.7 ± 15.2	2.9 ± 13.6	1.7 ± 14.6
	Function in daily living (ADL)	5.9 ± 19.6	4.0 ± 16.5	1.4 ± 13.2
	Function in sport and recreation (sport/rec)	10.5 ± 18.0	4.2 ± 16.7	2.3 ± 15.4
	Knee-related quality of life (QoL)	5.6 ± 14.8	3.1 ± 14.9	− 0.2 ± 16.2
KOOS change at T3 (difference between T3 and baseline)	*N*	47	38	40
	Global score	56.3 ± 82.6	48. ± 73.1	42.1 ± 66.2
	Pain	12.3 ± 19.4	12.8 ± 18.4	10.8 ± 16.5
	Symptoms	10.0 ± 16.6	7.4 ± 16.0	7.5 ± 14.7
	Function in daily living (ADL)	9.2 ± 19.5	10.3 ± 20.9	7.3 ± 14.6
	Function in sport and recreation (sport/rec)	11.1 ± 20.5	9.6 ± 15.3	9.7 ± 17. 8
	Knee related quality of life (QoL)	12.4 ± 20.3	9.2 ± 19.3	6.6 ± 16.8

**Fig. 5:**
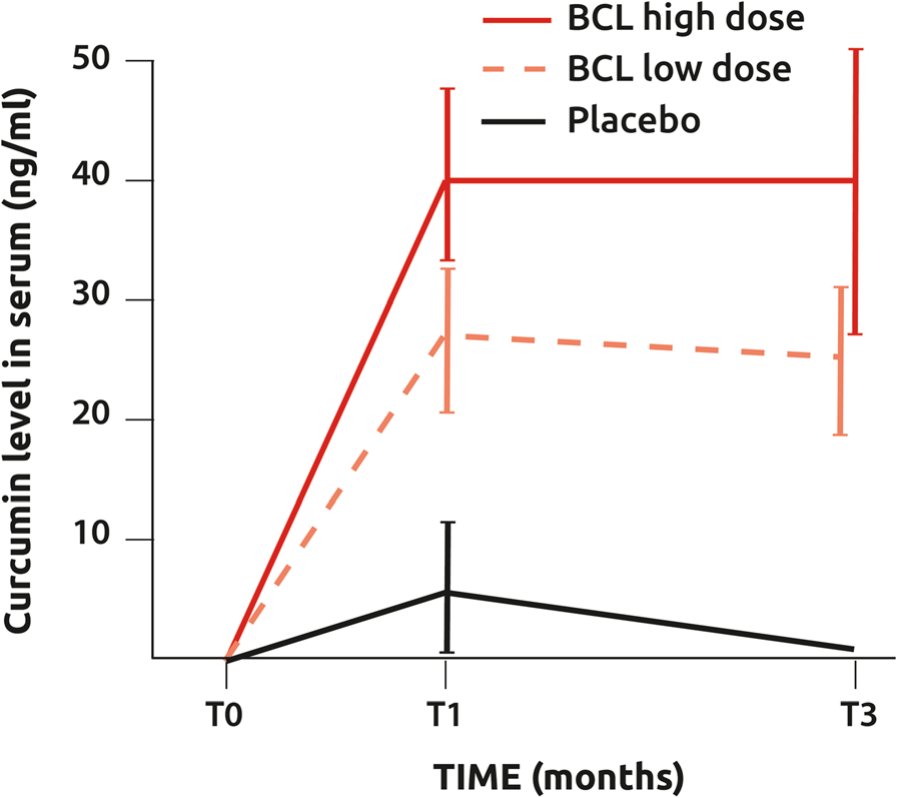
Curcumin levels in the serum of patient receiving daily doses of BCL or placebo

Study of compound usage was > 80% in all groups and comparable in the placebo and BCL groups, demonstrating excellent compliance. This was confirmed by curcumin measurement in PP. Curcumin serum levels dramatically increased at T1 (*p* < 0.001) and then remained stable until T3 in the low BCL group (*p* = 0.639) while undetectable in the placebo group (*p* = 0.948)(Fig. 5). At T1, but not at T3, curcumin levels were significantly higher in BCL high dose than in BCL low dose (*p* = 0.034).

Finally, there were significantly more adverse events (AE) related to the product in the BCL high dose than in the BCL low dose and placebo (37%, 21%, 13%, respectively, *p* = 0.012). No difference between BCL low dose and placebo was observed. There were no more AE leading to withdrawal in the BCL groups than in placebo. Abdominal discomfort and diarrhea were the most frequently reported AE. Routine laboratory testing did not identify any abnormalities in hepatic or renal dysfunction.

The mean paracetamol or NSAID intake decreased with time in all groups, but no significant difference was observed between the groups at different time points. While global analyses did not reveal any significant difference between the groups at different time points, complementary analyses on FAS population, covering within-group difference, revealed that only subjects of the BCL high-dose group showed a significant decrease in the intake frequency of paracetamol at different time points (*p* = 0.031 at T1, *p* = 0.0016 at T3) while no variation with time was observed in the low-dose and placebo group. For NSAIDs, a significant decrease of intake frequency was observed only in the low-dose group (*p* = 0.038 at T1, *p* = 0.029 at T3).

## Discussion

In this paper, we report the data of a phase II clinical trial investigating the clinical efficacy of two doses of a bio-optimized curcuminoids extract, administered orally during 3 months in patients with symptomatic established knee OA. To our knowledge, this is the first study supporting some therapeutic effect of bio-optimized curcumin in knee OA in a well-designed prospective multicenter trial. Indeed, some recent meta-analyses have pointed out the poor quality of clinical trials investigating food supplements including curcuminoids extracts [20, 21]. The data indicate that BCL, as an adjuvant treatment to paracetamol and/or NSAIDs, is superior to placebo to decrease pain and PGADA from the first month of treatment. The improvement in pain observed in the BCL low-dose group corresponds to an effect size (ES) of 0.35 for pain at T1 and 0.43 at T3. ES ≤ 0.2 is usually considered as small while ES between 0.2 and 0.5 is defined as medium. This corroborates the results of previous studies suggesting beneficial effects of curcumin on pain in rheumatoid arthritis [22]. This analgesic effect is all the more remarkable as it is observed in patients who used paracetamol and NSAIDs before and during the study. This means that in our population, BCL has an analgesic effect on top of the expected effect of paracetamol and NSAIDs. Interestingly, NSAID consumption decreased in the low-dose group over time, suggesting that at low dose, BCL decreases pain and NSAID consumption. Considering the adverse effects of NSAIDs, mainly in patients with co-morbidities, BCL could be of value as an alternative to NSAIDs in OA treatment [27, 28]. Surprisingly, no significant difference was observed with the KOOS. This can be explained by the fact that KOOS was evaluated on a 5-point Likert scale while knee pain was recorded using a visual analog visual scale of 100 mm which has a better ability to detect clinical change than the Likert scale [29].

One strength of this study was that curcumin levels have been measured in the blood of subjects to evaluate the compliance and the bioavailability of the product. Curcumin levels raised rapidly in the blood of all treated patients while remained undetectable in the placebo group. Another key observation was that the molecule reached a steady state after 1 month. In addition, plateau curcumin levels were comparable in the high- and low-dose BCL groups. This finding provides some indication of why both doses showed similar effects on pain. This, combined with the observation that the number of adverse effects related to the product was increased in the high BCL group, justifies the use of low-dose BCL in OA management. This also explains the higher drop-out in the high BCL group. The observed adverse effects were mainly diarrhea. In pharmacological terms, curcumin is a complete choleretic-cholagogue. The cleavage products of curcumin (feluric and hydrofeluric acids) have cholecistokinetic properties because they squeeze the gallbladder, while another principle product, para-tolilmethilcarbinol, has a strong choleretic activity. The choleretic effect of curcumin increases bile production by approximately 62% [30].

Post hoc analyses have also demonstrated that serum Coll2-1, a biomarker of type II collagen degradation, decreased in the BCL groups while slightly increased in the placebo group [24, 25]. Of course, this observation has to be verified in a larger population, and it remains to be demonstrated that this is associated with changes in structural parameters by imaging but suggest that BCL could downregulate cartilage catabolism and could therefore slow down structural progression.

This study showed some limitations. The main one is the small sample size. It is clear that larger sample sizes are required to confirm the positive trends observed in this study. The second limitation was the particularities of the study population. Indeed, the study population included patients with continuous pain despite the frequent intake of paracetamol and/or NSAIDs thus predominantly non-responders to standard OA pain treatment and thus not very sensitive to anti-inflammatory treatment.

## Conclusions

In conclusion, this study provided evidence that daily intake of 186.6 mg/day of BCL in patients with symptomatic knee OA leads to a reduction in pain superior to placebo with a good safety profile and a good compliance, despite the use of paracetamol and/or NSAIDs. Moreover, this trial provides useful information for the design of a larger phase III clinical trial including the sample size estimate, the choice of the dose, and the selection of primary outcomes.

## Additional files

Additional file 1: **Table S1.** FAS population changes between the follow-up visits (T1, T3, and T6) and baseline and efficacy analysis: results from repeated measures ANCOVA model with post hoc tests if significant. (DOCX 33 kb)

Additional file 2: Statistical analysis plan. (PDF 1655 kb)

## Data Availability

All the data are available and can be requested at Tilman SA (Baillonville, Belgium, yd@tilman.be).

## Abbreviations

ACR: American College of Rheumatology
AE: Adverse events
BCL: Bio-optimized *Curcuma longa* extracts
ELISA: Enzyme-linked immunosorbent assay
ES: Effect size
FAS: Full analysis set
ICH: International Council for Harmonization
ITT: Intention to treat
KOOS: Knee injury and Osteoarthritis Outcome Score
NF-κβ: Nuclear factor-kappa β
NSAID: Non-steroidal anti-inflammatory drug
OA: Osteoarthritis
PGADA: Patient Global Assessment of Disease Activity
SP: Safety population
SYSADOA: Symptomatic slow-acting drugs in OA
VAS: Visual analog scale
