# Supplementary material for: Bio-optimized Curcuma longa extract is efficient on knee osteoarthritis pain: a double-blind multicenter randomized placebo controlled three-arm study
Source: Arthritis Res Ther. 2019 Jul 27;21:179. doi: 10.1186/s13075-019-1960-5 (PMC6661105; doi:10.1186/s13075-019-1960-5)
Supplement: Supplementary file 1 — Table S1. FAS population changes between the follow-up visits (T1, T3, and T6) and baseline and efficacy analysis: results from repeated measures ANCOVA model with post hoc tests if significant. (DOCX 33 kb) [file 13075_2019_1960_MOESM1_ESM.docx]

| **Table S1: - FAS population-**  **Changes between the follow-up visits (T1, T3 and T6) and baseline &**  **Efficacy analysis: results from repeated measures ANCOVA model with post-hoc tests if significant** | | | | | | |
| --- | --- | --- | --- | --- | --- | --- |
|  | | | **BCL high dose**  **Mean (SD)** | **BCL low dose**  **Mean (SD)** | **Placebo**  **Mean (SD)** | **Treatment effect** |
| **Biomarker**  **sCOLL2-1** | Change at T1 | N | 49 | 44 | 44 | NS |
|  |  | Level in nM | -13.43 (117.667) | 15.76 (102.866) | 28.26 (131.666) |  |
|  | Change at T3 | N | 47 | 36 | 40 |  |
|  |  | Level in nM | -25.21 (129.035) | -22.67 (106.010) | -2.30 (97.535) |  |
|  | Change at T6 | N | 34 | 31 | 35 |  |
|  |  | Level in nM | -28.77 (134.951) | 19.42 (135.441) | 1.74 (77.175) |  |
|  | **Time effect: NS** | | NS | | | NS |
| **Global assessment of disease activity (PGADA)** | Change at T1 | N | 49 | 46 | 45 |  |
|  |  | VAS in mm | -14.49 (27.874) | -16.70 (24.870) | -4.22 (23.236) | NS |
|  | Change at T3 | N | 48 | 38 | 41 |  |
|  |  | VAS in mm | -23.31 (30.990) | -25.68 (30.307) | -11.41 (27.503) |  |
|  | Change at T6 | N | 37 | 34 | 37 |  |
|  |  | VAS in mm | -23.95 (31.339) | -28.03 (35.446) | -23.16 (27.498) |  |
|  | **Time effect: p<0.001** | | **Post-hoc test: P<0.001 for**  **T0 vs T1, T0 vs T3 and T0 vs T6** | | | NS |
| **Ultrasensitive CRP** | Change at T1 | N | 48 | 45 | 43 |  |
|  |  | Level in mg/L | -0.04 (1.421) | 0.07 (2.250) | 0.03 (2.834) | NS |
|  | Change at T3 | N | 47 | 38 | 40 |  |
|  |  | Level in mg/L | 0.34 (2.242) | -0.50 (2.387) | 0.20 (2.830) |  |
|  | Change at T6 | N | 35 | 32 | 35 |  |
|  |  | Level in mg/L | -0.05 (1.537) | -0.15 (2.340) | -0.43 (2.412) |  |
|  | **Time effect: NS** | | NS | | | NS |
| **Mean Knee Pain of the last 24 hours** | Change at T1 | N | 48 | 46 | 45 | NS |
|  |  | VAS in mm | -16.69 (25.191) | -17.93 (23.039) | -6.53 (19.405) |  |
|  | Change at T3 | N | 48 | 38 | 40 |  |
|  |  | VAS in mm | -25.04 (26.985) | -28.00 (28.176) | -12.25 (26.249) |  |
|  | Change at T6 | N | 37 | 34 | 37 |  |
|  |  | VAS in mm | -28.30 (26.817) | -23.00 (30.569) | -23.19 (26.471) |  |
|  | **Time effect: p<0.001** | | **Post-hoc test:** | | | **Time*Treatment effect: p<0.001** |
|  |  |  | **P<0.001 for**  **T0 vs T1, T0 vs T3 and T0 vs T6** | **P<0.001 for**  **T0 vs T1, T0 vs T3 and T0 vs T6** | **P<0.001 for**  **T0 vs T6** |  |
| **KOOS - Global score** | Change at T1 | N | 46 | 42 | 43 | NS |
|  |  | Score | 35.16 (67.451) | 18.02 (57.618) | 7.97 (60.193) |  |
|  | Change at T3 | N | 45 | 36 | 38 |  |
|  |  | Score | 56.34 (82.663) | 48.75 (73.139) | 42.10 (66.223) |  |
|  | Change at T6 | N | 37 | 33 | 37 |  |
|  |  | Score | 93.54 (103.282) | 56.05 (102.470) | 74.66 (83.293) |  |
|  | **Time effect: p<0.001** | | **Post-hoc test: P=0.012 for T0 vs T1 and**  **P<0.001 for T0 vs T3 and T0 vs T6** | | | NS |
| **KOOS - Pain** | Change at T1 | N | 48 | 45 | 45 | NS |
|  |  | Score | 7.15 (17.468) | 4.81 (16.735) | 3.10 (13.862) |  |
|  | Change at T3 | N | 47 | 38 | 39 |  |
|  |  | Score | 12.33 (19.396) | 12.80 (18.420) | 10.80 (16.504) |  |
|  | Change at T6 | N | 38 | 34 | 38 |  |
|  |  | Score | 19.23 (22.639) | 10.71 (24.661) | 17.53 (19.239) |  |
|  | **Time effect: p<0.001** | | **Post-hoc test: P=0.001 for T0 vs T1 and**  **P<0.001 for T0 vs T3 and T0 vs T6** | | | NS |
| **KOOS - Symptoms** | Change at T1 | N | 49 | 46 | 45 | NS |
|  |  | Score | 6.71 (15.216) | 2.86 (13.647) | 1.69 (14.590) |  |
|  | Change at T3 | N | 47 | 38 | 40 |  |
|  |  | Score | 10.03 (16.671) | 7.42 (16.045) | 7.51 (14.765) |  |
|  | Change at T6 | N | 38 | 34 | 38 |  |
|  |  | Score | 14.24 (17.171) | 10.00 (19.849) | 12.97 (16.781) |  |
|  | **Time effect: p<0.001** | | **Post-hoc test: P=0.007 for T0 vs T1 and**  **P<0.001 for T0 vs T3 and T0 vs T6** | | | NS |
| **KOOS – Function in daily living (ADL)** | Change at T1 | N | 48 | 46 | 45 | NS |
|  |  | Score | 5.89 (19.566) | 4.00 (16.532) | 1.41 (13.211) |  |
|  | Change at T3 | N | 47 | 38 | 40 |  |
|  |  | Score | 9.23 (19.501) | 10.34 (20.945) | 7.31 (14.608) |  |
|  | Change at T6 | N | 38 | 34 | 38 |  |
|  |  | Score | 16.44 (22.367) | 12.41 (23.723) | 13.82 (20.158) |  |
|  | **Time effect: p<0.001** | | **Post-hoc test: P<0.001 for T0 vs T3 and T0 vs T6** | | | NS |
| **KOOS - Function**  **in sports and recreation (Sport/Rec)** | Change at T1 | N | 47 | 43 | 43 | NS |
|  |  | Score | 10.49 (18.097) | 4.21 (16.777) | 2.28 (15.406) |  |
|  | Change at T3 | N | 45 | 36 | 39 |  |
|  |  | Score | 11.13 (20.492) | 9.66 (15.278) | 9.72 (17.769) |  |
|  | Change at T6 | N | 37 | 33 | 37 |  |
|  |  | Score | 22.76 (29.816) | 13.09 (23.908) | 16.32 (20.369) |  |
|  | **Time effect: p<0.001** | | **Post-hoc test: P=0.003 for T0 vs T1 and**  **P<0.001 for T0 vs T3 and T0 vs T6** | | | NS |
| **KOOS – Knee related Quality of Life (QoL)** | Change at T1 | N | 49 | 46 | 45 | NS |
|  |  | Score | 5.61 (14.837) | 3.13 (14.951) | -0.23 (16.212) |  |
|  | Change at T3 | N | 47 | 38 | 40 |  |
|  |  | Score | 12.37 (20.336) | 9.21 (19.372) | 6.61 (16.786) |  |
|  | Change at T6 | N | 38 | 34 | 38 |  |
|  |  | Score | 17.93 (25.633) | 11.03 (23.338) | 14.53 (19.097) |  |
|  | **Time effect: p<0.001** | | **Post-hoc test: P<0.001 for T0 vs T3 and T0 vs T6** | | | NS |
| **Pill Count** | Non-significant differences between groups (Kruskal Wallis test) | | | | | |
| **Curcumin level**  **in serum**  **(7 metabolites)** | Time, treatment and time*treatment are significant (p<0.001). Post-hoc test: Placebo vs BCL high dose and Placebo vs BCL low dose are significantly different at all timepoint (p<0.006). BCL high dose and BCL low dose are significantly different at T1 (p=0.034). For both treated groups, T0 vs T1, T0 vs T3 and T0 vs T6 are significant (p<0.001). | | | | | |
| **Patient’s satisfaction with treatment (Likert scale)** | Time, treatment and time*treatment are non-significant | | | | | |
| **Use of oral rescue treatments for knee pain in the last month** | Time, treatment and time*treatment are non-significant | | | | | |
|  | | | | | | |
| NS, non-significant; SD, standard deviation | | | | | | |
