# Supplementary material for: Bio-optimized Curcuma longa extract is efficient on knee osteoarthritis pain: a double-blind multicenter randomized placebo controlled three-arm study
Source: Arthritis Res Ther. 2019 Jul 27;21:179. doi: 10.1186/s13075-019-1960-5 (PMC6661105; doi:10.1186/s13075-019-1960-5)
Supplement: Supplementary file 2 — Statistical analysis plan. (PDF 1655 kb) [file 13075_2019_1960_MOESM2_ESM.pdf]

Statistical Analysis Plan

Evaluation of efficacy, tolerance, and dose effects of a Curcuma extract (FLEXOFYTOL®) versus PLACEBO in patients with knee osteoARthritis (COPRA)

STUDY CODE: COPRA

**Protocol Number:**	V6.1 – Amendment 2
**Test Drug:**	FLEXOFYTOL® (curcuma extract)
**Sponsor:**	Tilman SA
**Date:**	
**Version:**	Version 01

Confidential

NAME AND ADDRESSES

The signatories agree to comply in all respects with this statistical analysis plan.

	NAME / ADDRESS
**SPONSOR**<br><br><br><br><br><br><br><br>Sponsor's legal representatives	**Tilman SA**<br>Zoning Industriel Sud<br>B-5377 Baillonville, Belgium<br><br>Contact :<br>Email:<br>Tel : +32 (0)84 320 360<br><br>Yvan Dierckxsens<br>Email: yd@tilman.be<br>Office: +32 84 320 360
**CLINICAL CRO**<br><br><br><br><br><br><br><br><br>CRO's legal representatives	**ARTIALIS, S.A.**<br>Avenue de l'Hôpital, 11, Tour Giga B34<br>B-4000 LIEGE, Belgium<br><br>Contact:<br>Bérénice Costes, Ph.D.<br>Email: berenice.costes@artialis.com<br>Office: + 32 4 242 77 36<br><br>Pr. Yves Henrotin<br>Email: yves.henrotin@artialis.com<br>Office: +32 4 242 77 27
**BIOSTATISTICS**<br><br><br><br><br>CRO's legal representatives	**SOLADIS**<br>6 et 8 Rue Bellecombe<br>69006 LYON, France<br><br>Contact:<br>Frédéric Chavanel<br>Email: fchavanel@soladis.fr<br>Office : +33 4 72 838 678

APPROVALS

Sponsor's Representatives:

Yvan Dierckxsens

Company: **TILMAN**

Signature:

....

Date: __/___/____

Artialis' representative

Pr. Yves Henrotin

Signature:

....

Date: __/___/____

Statistician: Frédéric CHAVANEL

Company: **SOLADIS**

Signature:

....

Date: 20/09/2017

APPROVALS

Sponsor's Representatives:

Yvan Dierckxsens

Company: **TILMAN**

Signature: ....

Date: 20/09/2017

Artialis' representative

Pr. Yves Henrotin

Signature:

....

Date: __/___/____

Statistician:

Company: **SOLADIS**

Signature:

....

Date: __/___/____

APPROVALS

**Sponsor's Representatives**:	
	Yvan Dierckxsens
Company:	**TILMAN**
Signature:	
	....
Date:	_ _/ _ _ _/ _ _ _ _

**Artialis' representative**	
	Pr. Yves Henrotin
Signature:	
	....
Date:	_ _/ _ _ _/ _ _ _ _

**Statistician:**	
Company:	**SOLADIS**
Signature:	
	....
Date:	_ _/ _ _ _/ _ _ _ _

TABLE OF CONTENTS

Contenu

TABLE OF CONTENTS....4
1. ABBREVIATIONS 6
2. INTRODUCTION....7
3. PURPOSE....7
4. STUDY OBJECTIVES AND ENDPOINTS 7
4.1. Study Objectives 7
4.2. Study Endpoints (Target Variables)....8
4.2.1. Primary Target Variables....8
4.2.2. Secondary Target Variable(s)....8
4.3. Safety and tolerability....8
5. STUDY METHODS 9
5.1. Overall Study Design and Plan....9
5.2. Selection of Study Population....10
5.2.1. Inclusion criteria....11
5.2.2. Exclusion criteria 11
5.2.3. Withdrawal criteria....12
5.3. Method of Treatment Assignment and Randomization....12
5.4. Treatment Masking (Blinding)....12
6. SEQUENCE OF PLANNED ANALYSES 13
6.1. Interim Analyses....13
6.2. Final Analyses and Reporting....13
7. SAMPLE SIZE ESTIMATION....13
8. ANALYSIS POPULATIONS 14
9. STATISTICAL ANALYSIS 15
9.1. GENERAL PRINCIPLES....15
9.1.1. Analysis Software....15
9.1.2. Methods for Withdrawals, Missing Data, and Outliers 15
9.1.3. Data Transformations....16
9.1.4. Derived and Computed Variables....16
9.2. STUDY SUBJECTS 18
9.2.1. Disposition of Subjects and Withdrawals....18
9.2.2. Protocol Violations and Deviations 19

9.3. DEMOGRAPHICS AND OTHER BASELINE CHARACTERISTICS 22
9.3.1. Demographics 22
9.3.2. Prior and Concomitant Medications 23
9.3.3. Baseline Conditions 24
9.4. EFFICACY ANALYSES 29
9.4.1. Target variables 29
9.4.2. Methods of Efficacy analysis 30
9.4.3. Efficacy Descriptive Analysis 30
9.4.4. Treatment response - Primary Efficacy Variables 33
9.4.5. Treatment Response - Secondary Efficacy Variables 35
9.5. SAFETY AND TOLERABILITY ANALYSES 40
9.5.1. Adverse events 40
9.5.2. Pregnancies 43
9.5.3. Clinical Laboratory Evaluations 43
9.6. PATIENTS LISTING 45
9.7. COMPLEMENTARY EXPLORATORY ANALYSES 46
9.7.1. General Aspects 46
9.7.2. Statistical analysis – Specific aspects 46
10. REPORTING CONVENTIONS 47
10.1. General Reporting Conventions 47
10.2. Population Summary Conventions 47
10.3. Biomarker Related Conventions 48
10.4. Rounding 48
11. REFERENCES 48
12. TABLES, FIGURES AND GRAPHS 48
12.1. Summary Tables and Figures 48
12.2. Data Listings 53

ARTIALIS S.A., 11 Avenue de l'Hôpital, Sart-Tilman, tour GIGA, 4000 LIEGE – Belgium

Tel : 0032(0)4 2427706 – Fax : 0032(0)4 2427728

1. ABBREVIATIONS

AE	Adverse Event
ANOVA	Analysis of variance
Coll2-1	Type II collagen fragment
COPRA	Curcuma flexOfytol®Placebo osteoARthritis
CRF	Case Report Form
CSR	Clinical Study Report
e.g.	Exempli Gratia
FAS	Full Analysis Set
h	Hour
ICH	International Conference on Harmonization
NSAID	NonSteroidal Anti-Inflammatory Drug
IP	Investigational Product
ITT	Intended To Treat Population
KOOS	Knee Osteoarthritis Outcome Score
LOCF	Last observation Carried Forward
MD	Missing Data
mg	Milligram
N, n	Number
OA	OsteoArthritis
OARSI	OsteoArthritis Research Society International
OMERACT	Outcome Measures in Rheumatology
PP	Per Protocol Population
SAE	Serious Adverse Event
SAP	Statistical Analysis Plan
SD	Standard Deviation
SOP	Standard Operating Procedure
SP	Safety Population
t	Time
VAS	Visual Analogue Scale

2. INTRODUCTION

This Statistical Analysis Plan (SAP) describes the planned analysis and reporting for clinical research study COPRA "Evaluation of efficacy, tolerance, and dose effects of a Curcuma extract (FLEXOFYTOL®) versus PLACEBO in patients with knee osteoARthritis (COPRA)".

This study is being completed to evaluate the efficacy of two different dosages of FLEXOFYTOL® versus PLACEBO on arthritis-related and inflammatory biomarkers, on pain and on function in patients suffering from knee OA

The following documents were reviewed in preparation of this SAP:

- Clinical Trial Protocol COPRA, version V6.1 – Amendment 2
- Artialis' SOP "SAP, programing and statistical analysis", SOP-CLI-010.
- ICH Guidance Topic E9 "Statistical Principles for Clinical Trials" [1].

3. PURPOSE

The purpose of this SAP is to outline the planned analyses to be completed to support the completion of the Clinical Study Report (CSR) for the Clinical Trial Protocol COPRA, version 6.1 Amendment 2, of the clinical research study COPRA "Evaluation of efficacy, tolerance, and dose effects of a Curcuma extract (FLEXOFYTOL®) versus PLACEBO in patients with knee osteoARthritis (COPRA)". The planned analyses identified in this SAP will be included in regulatory submissions and/or future manuscripts.

Of note, complementary analyses not necessarily identified in this SAP may be performed to support the clinical development program. Any post-hoc, or unplanned analyses not identified in this SAP performed will be clearly identified in the respective CSR.

4. STUDY OBJECTIVES AND ENDPOINTS

4.1. Study Objectives

Primary objective

Evaluate the efficacy of two different dosages of FLEXOFYTOL® versus PLACEBO on arthritis-related biomarker coll2-1 and on patient assessment of disease activity in patients suffering from knee OA after a 3 months treatment

Secondary objectives

- Evaluate the efficacy of two different dosages of FLEXOFYTOL® versus PLACEBO on arthritis-related and inflammatory biomarkers
- Evaluate the efficacy of two different dosages of FLEXOFYTOL® versus PLACEBO on pain and function
- Evaluate the tolerance, the compliance and the patients' satisfaction
- Evaluate the use of rescue treatments i.e. Paracetamol and oral non-steroidal anti-inflammatory drugs (NSAIDs) during the study

4.2. Study Endpoints (Target Variables)

4.2.1. Primary Target Variables

- Variation of serum levels of sColl2-1 biomarker between T0 and T3
- Variation of global assessment of disease activity using a VAS between T0 and T3

4.2.2. Secondary Target Variable(s)

- Variation of serum levels of sColl2-1 and ultrasensitive CRP biomarkers between T0 and the different time points

- Variation of pain and function between T0 and the different time points :
    - Mean knee pain over the last 24 hours using a visual analogue scale (VAS)
    - Global assessment of disease activity using a VAS
    - Knee injury and Osteoarthritis Outcome Score (KOOS) index and its subscale scores using a self-administered questionnaire. The KOOS outcome score is a 42-item self-administrated questionnaire comprising 5 subscales: Pain, Symptoms, Activities of Daily Living, Sports and Recreation function, and Quality Of Life. A separate score ranging from 0 to 100, where 100 represents the best result, will be calculated for each subscale. A global score will be calculated by summing the score of each subscale.

- Tolerance assessed at each follow-up visit as the incidence of adverse events and as the incidence of drop-outs. In case of adverse event, further blood safety (hepatic and renal function) will be performed.

- Compliance assessed by counting the capsules within the investigation kits brought back by the patient at each follow-up visit and by curcumin level monitoring in serum samples.

- Patients' satisfaction with treatment evaluated by the investigator at each follow-up visit by a five-category scale i.e. Better, little better, same, little lower or far lower

- The use of rescue treatments i.e. Paracetamol and oral NSAIDs during the month prior to each visit

4.3. Safety and tolerability

Adverse Events (AEs) and Serious Adverse Events (SAEs) occurring during the study will be documented in the CRF at each study visit, in order to assess the safety of product.

5. STUDY METHODS

5.1. Overall Study Design and Plan

**Trial design**	Prospective, multi-center, randomized, double-blind, placebo-controlled, 3 parallel-groups
**Investigational product**	FLEXOFYTOL® (curcuma extract)<br>Active ingredient : Turmeric rhizome extract (*Curcuma longa* L.) : 46.67 mg per cap<br>IP status : Dietary supplement (n°PL 31/100; Federal Public Service, Health, Food chain safety and environment)
**Comparator**	PLACEBO
**Dosage, group**	Three parallel-groups receiving each 2x3 caps per day (6 caps per day)<br>**Group A**: FLEXOFYTOL® high dosage 2x3 caps/day<br>**Group B**: FLEXOFYTOL® low dosage 2x2 caps/day + Placebo 2x1 cap/day<br>**Group C**: PLACEBO 2x3 caps/day
**Investigators**	Private or public hospital rheumatologists or articular disease specialists located in Belgium.
**Trial duration**	Participants will be recruited during minimum a 3-month period. Each patient will be enrolled in the study for 6 months including 4 visits: Inclusion visit (T0), follow-up visits after 1 month (T1), 3 months (T3) and 6 months (T6).
**Trial procedures**	**Recruitment**<br>Any recruitment process proposed by the investigator will be used including the following:<br>Control visit: During a control visit, the investigator will ask potential eligible patients if they are willing to participate to the trial. If so, the investigator will check eligible criteria and will perform an inclusion visit<br>Patient registry: The investigator will check his patient registry to identify potential eligible patients and will invite them to an inclusion visit.

ARTIALIS S.A., 11 Avenue de l’Hôpital, Sart-Tilman, tour GIGA, 4000 LIEGE – Belgium

Tel : 0032(0)4 2427706 – Fax : 0032(0)4 2427728

Schematic representation of the study

	**Inclusion visit T0**	**Follow-up visit T1**	**Follow-up visit T3**	**Follow-up visit T6**
**General evaluation**				
Informed consent	X			
Inclusion and exclusion criteria	X			
Demographic data	X			
Medical history	X			
Analyze radiographs of the last 12 months	X			
OA history and OA treatment history	X			
Concomitant medications	X	X	X	X
**Clinical evaluation**				
Mean knee pain over the last 24 hours (VAS)	X	X	X	X
Global assessment of disease activity (VAS)	X	X	X	X
KOOS index (self-administered questionnaire)	X	X	X	X
**Investigational product**				
IP instruction and delivering	X	X	X	
**Tolerance**				
Adverse events		X	X	X
Drop-outs		X	X	X
**Compliance**				
Pill count		X	X	X
**Satisfaction**				
Satisfaction scale		X	X	X
**Rescue treatments**				
Use of Paracetamol and/or NSAIDs in the month prior to visit	X	X	X	X
**Biological sampling**				
Serum samples (for biomarkers analyses, blood safety (only if AE) and curcumin level monitoring)	X	X	X	X

5.2. Selection of Study Population

150 patients suffering from symptomatic knee OA will be randomized in 3 parallel groups of 50 patients.

5.2.1. Inclusion criteria

Patients must meet ALL of the following criteria:

- Male or female between the age of 45 and 80
- Uni- or bilateral femorotibial and/or femoropatellar knee OA
  - Responding to clinical and radiological criteria of ACR
  - Symptomatic for more than 6 months in the most painful knee
  - Radiological K&L grade II-III in radiographs from less than 12 months
- History of mean knee pain score evaluated on VAS (0-100) ≥ 4 in the last 3 months (based on patient OA history) including the last 24 hours (the most painful knee is considered)
- Regular users of Paracetamol and/or oral NSAIDs to manage OA knee pain (based on patient OA history)
- Able to follow the instructions of the study
- Having signed an informed consent

5.2.2. Exclusion criteria

Patients that meet AT LEAST one of the following criteria will be excluded:

Related to the OA pathology

- Chondromatosis or villonodular synovitis of the knee
- Recent trauma of the knee responsible of the symptomatic knee
- Articular disease resulting from articular dysplasia, aspectic osteonecrosis, acromegaly, Paget's disease, hemophilia, hemochromatosis….
- Inflammatory disease i.e. rheumatoid arthritis, gout and infectious arthritis
- Pathologies interfering with the evaluation of OA (radiculalgia in the lower limbs, arteritis…..)
- Prosthesis on the target knee

Related to treatments

- Treatments based on strontium ranelate, bisphosphonates, selective estrogen-receptor modulator (SERM) and parathormone (PTH)
- Corticosteroids or hyaluronan injection in the target knee in the last 3 months
- Oral corticotherapy ≥ 5mg/day in the last 3 months
- OA treatment based on curcuma extract in the last 3 months
- Arthroscopy in the last 3 months
- Anticoagulant (coumarinic compound) and heparin (contra-indication for FLEXOFYTOL®)
- Symptomatic slow-acting drugs (SYSADs) treatment (Chondroitin, diacerein, glucosamine, soy and avocado unsaponifiables) in the previous month
- An anticipated need for any forbidden OA treatments during the trial

Related to associated diseases

- Severe and uncontrolled diseases (liver or renal failure, lung/heart severe disease, tumor, HIV….)
- Obstruction of the bile ducts
- Allergy or contra-indication to curcumin, Paracetamol and/or oral NSAIDs
- Severe alteration of mobility enabling functional evaluation
- Anticipated need for any surgical or other invasive procedure during the trial including prosthesis in the target knee

Related to patients

- Artialis (study coordinator) or Tilman (Sponsor) 's employees
- Participation to a therapeutic clinical trial in the last 3 months
- Under guardianship or judicial protection
- Pregnancy, breastfeeding, planned conception
- Women without menopause or tubal ligation without contraception

5.2.3. Withdrawal criteria

- Patients' decision
- Investigators' decision following an AE
- Disease incompatible with the pursuing of the study or requiring a specific treatment incompatible with the study
- An inability or unwillingness to comply with the study protocol, and the protocol deviations are sufficient to jeopardize the integrity of the study
- Prosthesis in the target knee
- Pregnancy

5.3. Method of Treatment Assignment and Randomization

Central randomization was performed by Frédéric CHAVANEL (Soladis), taking into account the following features:

Randomization Information	General list	Back up list
Number of subject	150 (001 to 150)	30 (151 to 180)
Number of groups	3 (A, B, C)	3 (A, B, C)
Number of subjects in each treatments groups	50	10
Information on the randomization	IP number, Group, Kit Number and Block	IP number, Group, Kit Number and Block
Size of randomization block	6	3
Seed of the randomization	145987	8741269
Software used to generate the randomization	SAS 9.2® - English	SAS 9.3® - English
Date	June 19th 2014	September 7th 2015

5.4. Treatment Masking (Blinding)

To achieve the blinding, each group received 6 (2x3) caps per day as follow:

Each kit contained a unique identification code of the kits and the treatment group (A, B, C)

Group A: FLEXOFYTOL® high dosage 2x3 caps/day

Group B: FLEXOFYTOL® low dosage 2x2 caps/day + Placebo 2x1 cap/day
Group C: PLACEBO 2x3 caps/day

To achieve a good balance between the treatments (A, B, C) delivered to each center, investigation kits were shipped by a multiple of 3 (3 treatment groups (A, B and C)). The investigator was asked to distribute the same treatment group every four patients to achieve a good balance between the 3 treatment groups within this center.

6. SEQUENCE OF PLANNED ANALYSES

6.1. Interim Analyses

No interim analyses are planned for COPRA study.

6.2. Final Analyses and Reporting

All final, planned, analyses identified in the protocol and in this SAP will be performed only after the last patient has completed the study. A data review meeting will be held prior to database lock and completion of the final analyses.

In addition, no database may be locked, or analyses completed until this SAP has been approved.
Key statistics and study results will be made available following database lock and prior to completion of the final clinical study report (CSR).

Any, post-hoc, exploratory analyses completed to support planned study analyses, which were not identified in this SAP, will be documented and reported in appendices to the CSR. Any results from these unplanned analyses will also be clearly identified in the text of the CSR.

7. SAMPLE SIZE ESTIMATION

Sample size was estimated following recommendations and guidance on statistical principles for clinical trials (Sakpal, 2010; ICH E9) and considering results obtained in Tilflexy002 (table below).

Sample size estimation :

(D0-D84)	sColl2-1	VAS disease activity
Mean treated group	-44.4	-21
Mean group placebo (assumption)	0	0
SD	53	25
Distribution	Normal	Normal
Number of sides	2	2
Size per group for nominal power of 90%, alpha 0.05	32	31
Size per group for nominal power of 85%, alpha 0.05	27	27

Size per group for nominal power of 85%, alpha 0.025	32	32

Considering the hypotheses of a difference in Coll2-1 biomarker level of 44.4nmol/L with standard deviation of 53 and a difference in patient assessment of global disease activity of 21mm with standard deviation of 25, a level of significance of 2.5% (Type I error) to take into account multiplicity testing due to co-primary endpoints, a Power of 85% (Type II error), and two-tailed type of test, the estimated sample size is 32. To take into account the drop-out rate and potential lack of full compliance to the treatment in an aged population, the sample size per group was increased to 50.

8. ANALYSIS POPULATIONS

All patients who sign an informed consent and participate in any aspect of the study are to be accounted for in one or more analysis populations. At a minimum, demographic and baseline data for all patients who agree to participate in a study are to be identified and reported.

The following analysis populations are defined for the study:

Safety Population (SP)

The safety population includes all patients treated with at least one dose of the investigational product. The safety analysis will be based on the Safety Population.

Intention to Treat Population (ITT)

ITT population includes every subject who is randomized according to randomized treatment assignment. It ignores noncompliance, protocol deviations, withdrawal, and anything that happens after randomization.

Full Analysis Set Population (FAS)

FAS is the set of subjects that is as close as possible to the ideal implied by the intention-to-treat principle. It is derived from the set of all randomized subjects by minimal and justified elimination of subjects. FAS set comprises subjects who have one efficacy basal measure and at least one corresponding post-baseline efficacy measurement for one of the main efficacy variables, regardless of subsequent withdrawal from treatment or deviation from the protocol.

Per-Protocol Population (PP)

The per-protocol population includes all treated subjects who had not suffered any major protocol deviation. Major deviations will be one of the following:

- Deviations on inclusion and exclusion criteria
- No measure of Primary outcomes at least at inclusion and after IP treatment (either at T1, T3 or T6)
- Less than 70 days of IP treatment
- Compliance lower than 80%

- Concomitant use of forbidden treatments
- More than 7 days between end of IP treatment and collection of blood samples at the last visit

9. STATISTICAL ANALYSIS

9.1. GENERAL PRINCIPLES

Summary tables (descriptive statistics and/or frequency tables) will be provided by study product and globally for all variables, as appropriate.

Continuous variables will be summarized with descriptive statistics per group and globally (n, number of missing data, arithmetic mean, standard deviation, median, 1st and 3rd quartile, Minimum and Maximum).

Assessment of the normality of data will be performed with Shapiro-Wilk normality test when n≤50 and with Kolmogorov-smirnov when n >50.

In some case, logarithmic (natural logs) transformation will be used for achieving normal distribution of the data set. If the assumptions of the models cannot be reached, non-parametric Friedman's tests will be performed.

Categorical data will be assessed using percentages and frequency, per group and globally. Effects on categorical variable will be evaluated by bilateral Chi2 tests or bilateral Fisher exact test. Pairwise comparisons will be performed when found significant.

Ordinal variables will be assessed using percentages and frequency, per group and globally. To evaluate effects on ordinal variables, bilateral Kruskal-Wallis test will be performed.

Statistical significance is achieved at 95% confidence (p-value significance < 0.05).

9.1.1. Analysis Software

Data management will be performed using OpenClinica v 3.1.4.1.

The "Relevant Medical History", "Disease History", and the Adverse Event recorded during the study will be coded using MedDRA version 20.0. Medication will be coded using the ATC/DDD Index 2017 recommended by the WHO.

Statistical analysis will be performed with SAS® software Version 9.2 or later (SAS Institute, Cary, North Carolina, USA).

9.1.2. Methods for Withdrawals, Missing Data, and Outliers

A multiple imputation will be performed to handle missing data as sensitive analysis in the PP population for the treatment response of the primary efficacy variables. The imputation model will include all the covariates used for the analysis model, provided convergence is achieved. Each incomplete variable will be modelled using all others variables present in the imputation model. Because the response variables are count data, they will be imputed by Predictive Mean Matching (PMM), using the MI procedure. The coefficient k for this method is set to 5, and the random seed to 1234. In order to deal with both monotone and non-monotone missingness, PMM

will be used with the Fully Conditional Specification algorithm (FCS). The chain will go through at least 20 burn-in iterations for each imputation. At least 20 imputed datasets will be generated; this number would be increased until the relative efficiency is stabilized. Each of the imputed datasets will then be analyzed independently by the primary efficacy analysis. Finally, their respective results will be pooled by using the MIANALYZE procedure. (References: Schenker, N., and Taylor, J. M. G. (1996) "Partially Parametric Techniques for Multiple Imputation" Computational Statistics and Data Analysis 22:425–446. Brand, J. P. L. (1999) "Development, Implementation, and Evaluation of Multiple Imputation Strategies for the Statistical Analysis of Incomplete Data Sets" Ph.D. thesis, Erasmus University. Van Buuren, S. (2007) "Multiple Imputation of Discrete and Continuous Data by Fully Conditional Specification" Statistical Methods in Medical Research 16:219–242.)

Exclusion of a particular value as an outlier will be allowed only if both medically and statistically justified, except for Coll2-1 values that will be justified statistically only. When suspected, statistical outliers will be identified by performing Generalized ESD Test for Outlier by paying attention to masking effect for multiple outliers in large data sets. In case of outlier removal, statistical analysis will be performed for complete data set and for data set with outlier value(s) removed and differences will be discussed.

Definition of the methods for withdrawals, missing data and outliers may be refined during the data review meeting with the consequent SAP updating.

9.1.3. Data Transformations

Data derived from the study may be subjected to a logarithmic (natural logs) transformation before analysis if data appear to more closely meet the assumptions of a statistical inference procedure.

9.1.4. Derived and Computed Variables

- Age is computed from date of birth and date of baseline visit. The number of days between those two dates is calculated and then divided by 365.25 to give age in years: (date of baseline – date of birth) / 365.25

- "Duration of knee OA" is computed from date of first symptoms and date of baseline visit. The number of days between those two dates is calculated and then divided by 365.25 to give duration of knee OA in years: (date of baseline– date of first symptoms) / 365.25

  If day of first symptoms is unknown, then first day of the month is used. If month of first symptoms is unknown, January is used.

- Duration of follow up period is computed from date of off study and date of baseline visit: The number of days between those two dates is calculated as date of off study - date of baseline visit

- Time to onset of AE is computed from start date of AE and date of baseline visit: The number of days between those two dates is calculated as date of start date of AE - baseline visit

- BMI: BMI is weight in kilograms divided by height in meters squared

- Compliance assessed with the pill count method is evaluated in % of the estimated versus theoretical consumption: Compliance (%) = estimated number of caps taken divided by number of theoretical caps multiplied by 100; where:

- Estimated number of caps taken = given caps – remaining caps ; with
  - Given caps = 210 at T1; 210*2=420 at T3 and 210*3=630 at T6
- Number of theoretical caps = (stop date – start date + x + y) * 6 ; with

Compliance between T0 T1.

Start Date based on Inclusion Visit – T0 page 5/19 CRF. Instructions and recommendations field “5. // First capsule will be taken the day after the T0 visit in the morning: DATE: dd/mm/yyyy”

Stop Date based on Follow up visit- T1 page 10/19 CRF. Instructions and recommendations field “5. // First capsule for this period will be taken: DATE: dd/mm/yyyy” Morning / Evening

X Factor based on Follow up visit- T1 page 10/19 CRF. Instructions and recommendations field “5. // First capsule for this period will be taken: DATE: dd/mm/yyyy” Morning / Evening
If Morning Select, X Factor = 0
If Evening Selected, X Factor = 0.5

Y Factor = 0

Compliance between T1 T3.

Start Date based on Follow up visit- T1 page 10/19 CRF. Instructions and recommendations field “5. // First capsule for this period will be taken: DATE: dd/mm/yyyy” Morning / Evening

Stop Date based on Follow up visit- T3 page 14/19 CRF. Instructions and recommendations field “5. // First capsule for this period will be taken: DATE: dd/mm/yyyy” Morning / Evening

X Factor based on Follow up visit- T3 page 14/19 CRF. Instructions and recommendations field “5. // First capsule for this period will be taken: DATE: dd/mm/yyyy” Morning / Evening
If Morning Select, X Factor = 0
If Evening Selected, X Factor = 0.5

Y Factor based on Follow up visit- T1 page 10/19 CRF. Instructions and recommendations field “5. // First capsule for this period will be taken: DATE: dd/mm/yyyy” Morning / Evening
If Morning Select, Y Factor = 0
if Evening Selected, y Factor = -0.5

Compliance between T3 T6.

Start Date based on Follow up visit- T3 page 14/19 CRF. Instructions and recommendations field “5. // First capsule for this period will be taken: DATE: dd/mm/yyyy” Morning / Evening

Stop Date based on Follow up visit- T6 page 16/19 CRF. Visit Date dd/mm/yyyy

X Factor based on Follow up visit- T6 page 19/19 CRF. Prescription for blood collection, Date dd/mm/yyyy and Hour of collection hh:mm.
If Date Blood Collection = Date Visit T6,
if Hour of collection is <= 12:00, X Factor = 0

if Hour of collection is > 12:00, X Factor = 0.5
If Date Blood Collection > Date Visit T6, X Factor = 0.5

Y Factor based on Follow up visit- T3 page 14/19 CRF. Instructions and recommendations field "5. // First capsule for this period will be taken: DATE: dd/mm/yyyy" Morning / Evening
If Morning Select, Y Factor = 0
if Evening Selected, y Factor = -0.5

9.2. STUDY SUBJECTS

9.2.1. Disposition of Subjects and Withdrawals

The following must be presented:

- Total number of subjects screened (=subjects who gave informed consent).
- Number of included patients.
- In each arm, number of included patients who completed the study.
- In each arm, number of subjects who discontinued after inclusion in the study and main reason.

All subjects who signed the informed consent will be accounted for in this study.

	ALL (N=XXX)	Group A (N=XXX)	Group B (N=XXX)	Group C (N=XXX)
Screened subject	XXX			
Randomized subjects	XXX	XXX	XXX	XXX
Early withdrawal	XXX	XXX	XXX	XXX
Completed subjects	XXX	XXX	XXX	XXX

Table X: Patients disposition

Causes for study withdrawals will be described by the frequency and percent of subjects in each population.

	ALL (N=XXX)	Group A (N=XXX)	Group B (N=XXX)	Group C (N=XXX)
Causes of study withdrawals:				
- Xx	XX (X%)	XX (X%)	XX (X%)	XX (X%)
- Xx	XX (X%)	XX (X%)	XX (X%)	XX (X%)
- Xx	XX (X%)	XX (X%)	XX (X%)	XX (X%)
- Xx	XX (X%)	XX (X%)	XX (X%)	XX (X%)

Table X: Withdrawals reasons

Additionally to the tables above, other table will be performed in order to present the median follow-up period for each group and the number of included subjects per site by the frequency and percent.

The following flowchart will also be presented:

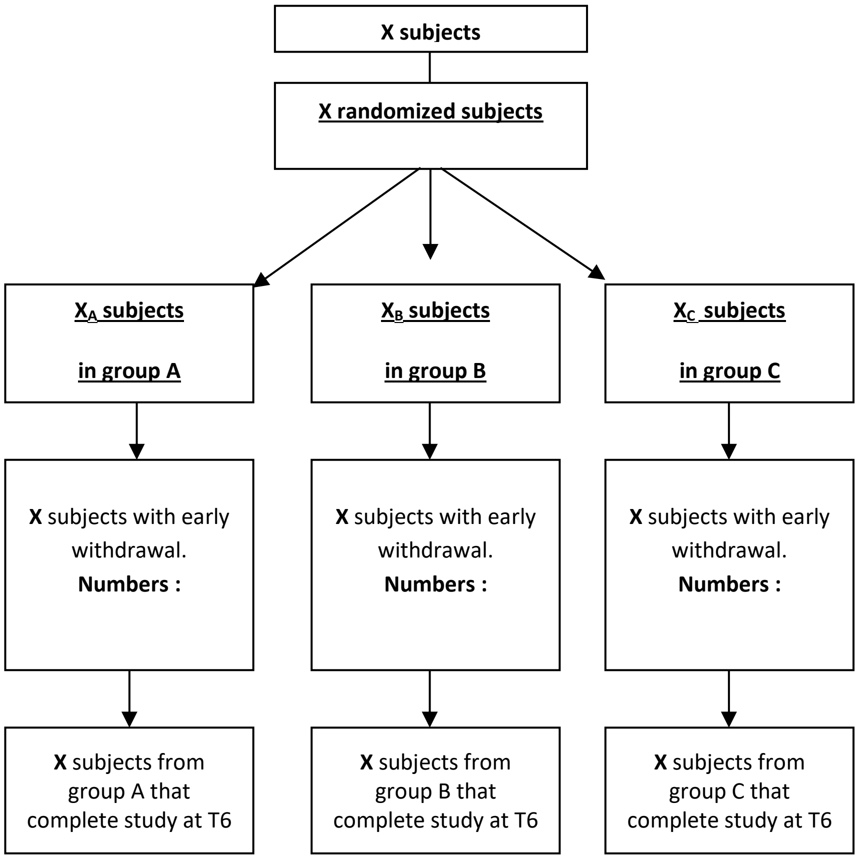

9.2.2. Protocol Violations and Deviations

The analysis populations defined in Section 8 will be summarized by treatment group and globally. Patients who do not finish the treatment period due to early withdrawal will still be included in the safety analysis.

	**ALL (N=XXX)**	**Group A (N=XXX)**	**Group B (N=XXX)**	**Group C (N=XXX)**
Screened subject	XXX			
Randomized subjects	XXX	XXX	XXX	XXX
SP population	XXX	XXX	XXX	XXX
ITT population	XXX	XXX	XXX	XXX
FAS population	XXX	XXX	XXX	XXX
PP population	XXX	XXX	XXX	XXX

Number of subjects with major violation and number of subjects with any minor protocol violation/deviation will be listed in the CSR.

Protocol violations and relative reasons will be also summarized by treatment group and globally.

	Group A	Group B	Group C	Total
Number of subjects that have had at least one protocol deviation	XX (100%)	XX (100%)	XX (100%)	XX (100%)
Number of subjects that have had at least one major protocol deviation	XX (100%)	XX (100%)	XX (100%)	XX (100%)
Causes of major deviations :<br>- Xx<br>- Xx<br>- Xx<br>- Xx	 XX (X%)<br>XX (X%)<br>XX (X%)<br>XX (X%)	 XX (X%)<br>XX (X%)<br>XX (X%)<br>XX (X%)	 XX (X%)<br>XX (X%)<br>XX (X%)<br>XX (X%)	 XX (X%)<br>XX (X%)<br>XX (X%)<br>XX (X%)

Table 1 Major protocol deviations

The following flowchart will also be presented:

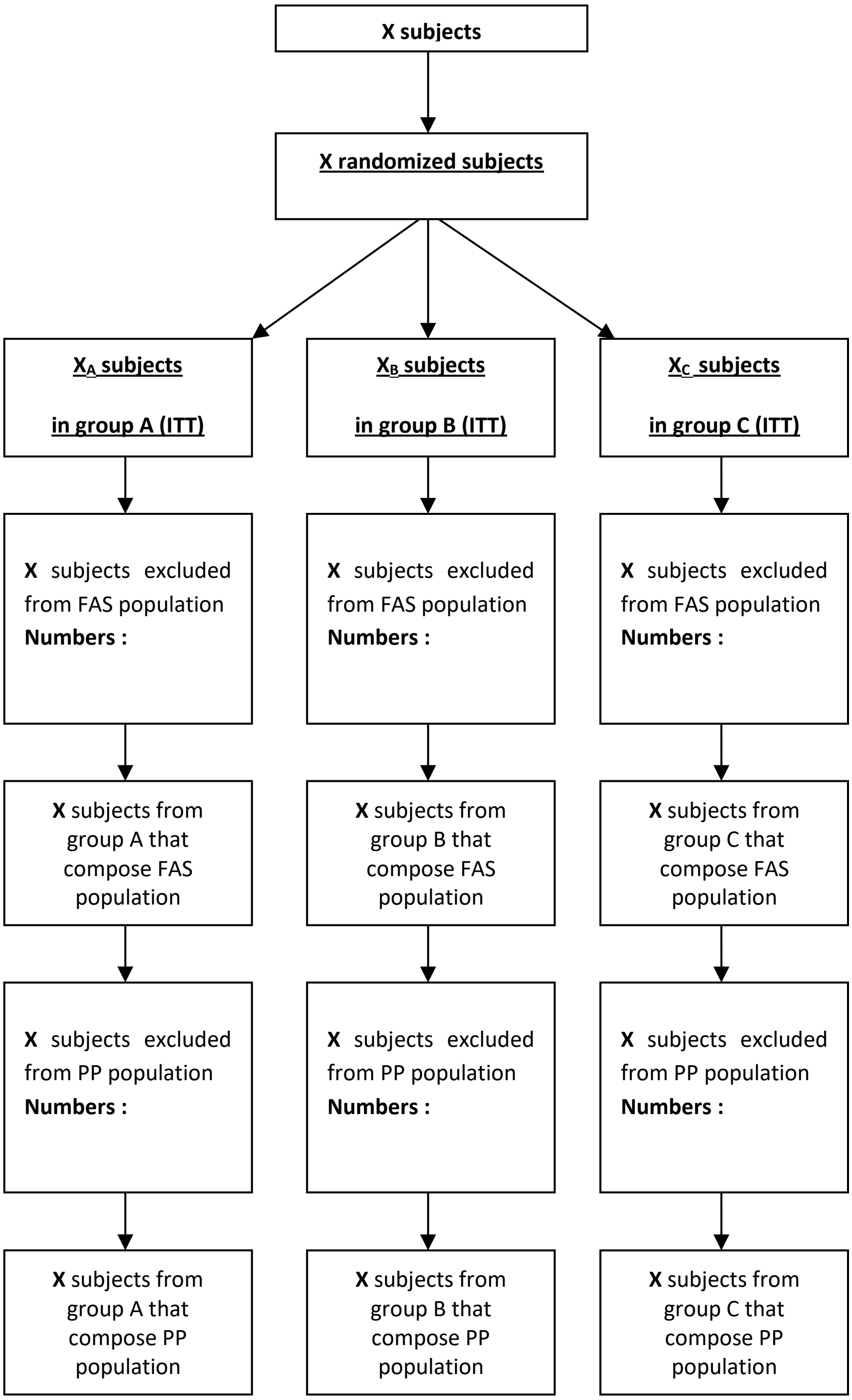

9.3. DEMOGRAPHICS AND OTHER BASELINE CHARACTERISTICS

The analysis of demographic and baseline characteristics of the study population is critical for describing its homogeneity or heterogeneity between groups.

Demographic data include, but are not limited to age, gender and baseline characteristics. Other characteristics records may include medically relevant previous disease(s) and surgeries, allergies, concomitant drug therapy (for not permitted medication), body weight and height, BMI, etc.

9.3.1. Demographics

Demographic characteristics will be summarized for both FAS and PP populations.

Continuous variables, namely age, weight, height and BMI will be summarized per group and globally, using N, number of missing data (MD), arithmetic mean, standard deviation, median, 1st and 3rd quartile, and min-max. Comparisons will be evaluated using ANOVA with Tukey Post hoc test. Model p-values will be reported. Assessment of the normality of data will be performed with Shapiro-Wilk normality test when n≤50 and with Kolmogorov-smirnov when n >50. If not Normal the analyses will be conducted on rank.

Categorical variables, namely gender, will be summarized using the frequency and percentage per group and globally. Comparisons will be evaluated using khi square tests. Model p-values will be reported. If significant, pairwise comparisons will be performed.

		ALL (N=XXX)	Group A (N=XXX)	Group B (N=XXX)	Group C (N=XXX)	P-Value
**Demographic factors**						
Age (years)	N (MD)	XXX (XXX)	XXX (XXX)	XXX (XXX)	XXX (XXX)	0.005 (S)§
	Mean (SD)	XX.X (X.X)	XX.X (X.X)	XX.X (X.X)	XX.X (X.X)	
	Median [Q1-Q3]	XX.X [XX.X; XX.X]	XX.X [XX.X; XX.X]	XX.X [XX.X; XX.X]	XX.X [XX.X; XX.X]	
	Min - Max	XXX - XXX	XXX - XXX	XXX - XXX	XXX - XXX	
BMI (Kg/m²)	N (MD)	XXX (XXX)	XXX (XXX)	XXX (XXX)	XXX (XXX)	0.005 (S)§
	Mean (SD)	XX.X (X.X)	XX.X (X.X)	XX.X (X.X)	XX.X (X.X)	
	Median [Q1-Q3]	XX.X [XX.X; XX.X]	XX.X [XX.X; XX.X]	XX.X [XX.X; XX.X]	XX.X [XX.X; XX.X]	
	Min - Max	XXX - XXX	XXX - XXX	XXX - XXX	XXX - XXX	
Gender	Male	XXX/XXX (XX.X%)	XXX/XXX (XX.X%)	XXX/XXX (XX.X%)	XXX/XXX (XX.X%)	<0.001(S)†
	Female	XXX/XXX (XX.X%)	XXX/XXX (XX.X%)	XXX/XXX (XX.X%)	XXX/XXX (XX.X%)	

† Chi-Square test
§ ANOVA test

Table 2 Demographic data at baseline. PP population

ARTIALIS S.A., 11 Avenue de l'Hôpital, Sart-Tilman, tour GIGA, 4000 LIEGE – Belgium

Tel : 0032(0)4 2427706 – Fax : 0032(0)4 2427728

If significant, Post hoc Tukey tests will be performed:

Comparison	Means difference	Adjusted P-value* (Significance)	Adjusted 95% CI
Group A vs. Group B	X.XXX	X.XXX (Not significant)	[X.XXX; X.XXX]
Group A vs. Group C	X.XXX	X.XXX **(Significant)**	[X.XXX; X.XXX]
Group B vs. Group C	X.XXX	X.XXX **(Significant)**	[X.XXX; X.XXX]

* Bilateral Tukey adjustment

Table 3 Demographic data at baseline. Pairwise comparisons. PP population

9.3.2. Prior and Concomitant Medications

All previous and concomitant therapies will be recorded and documented. For each group:

- Description of prior treatments by ATC name (frequency and percentage)Number of patients taking at least one concomitant treatment by ACT name Description of concomitant treatments by ATC name and molecule (frequency and percentage)

	ALL (N=XXX)	Group A (N=XXX)	Group B (N=XXX)	Group C (N=XXX)
Prior medication	XX	XX	XX	XX
ATC XXXXX	XX (X%)	XX (X%)	XX (X%)	XX (X%)
ATC XXXXX	XX (X%)	XX (X%)	XX (X%)	XX (X%)
ATC XXXXX	XX (X%)	XX (X%)	XX (X%)	XX (X%)

Table X: Prior medication by ATC molecule

	ALL (N=XXX)	Group A (N=XXX)	Group B (N=XXX)	Group C (N=XXX)
Patients taking at least one concomitant medication	XX	XX	XX	XX
ATC XXXXX	XX (X%)	XX (X%)	XX (X%)	XX (X%)
ATC XXXXX	XX (X%)	XX (X%)	XX (X%)	XX (X%)
ATC XXXXX	XX (X%)	XX (X%)	XX (X%)	XX (X%)

Table X: Number of patients taking at least one concomitant medication by ATC name

	ALL (N=XXX)	Group A (N=XXX)	Group B (N=XXX)	Group C (N=XXX)
Concomitant medication	XX	XX	XX	XX
ATC XXXXX	XX (X%)	XX (X%)	XX (X%)	XX (X%)
ATC XXXXX	XX (X%)	XX (X%)	XX (X%)	XX (X%)
ATC XXXXX	XX (X%)	XX (X%)	XX (X%)	XX (X%)

Table X: Concomitant medication by ATC name

	ALL (N=XXX)	Group A (N=XXX)	Group B (N=XXX)	Group C (N=XXX)
Concomitant medication	XX	XX	XX	XX
Molecule XXXXX	XX (X%)	XX (X%)	XX (X%)	XX (X%)
Molecule XXXXX	XX (X%)	XX (X%)	XX (X%)	XX (X%)
Molecule XXXXX	XX (X%)	XX (X%)	XX (X%)	XX (X%)

Table X: Concomitant medication by molecule

Medication will be coded using the ATC/DDD Index 2015 recommended by the WHO.

9.3.3. Baseline Conditions

The analysis of baseline characteristics of the study population is critical for describing relevant illness (medical history) and concomitant illness at the initiation of a patient’s participation. Relevant co-morbidities should be highlighted and particular attention in describing the findings for these conditions should be the emphasis of this section. These summaries will be presented for the Safety Population.

Baseline Medical History

Summaries of the presence of significant medical history at baseline will be presented for the Safety Population.

- Number of patients with significant medical/surgical history continuing/not continuing at baseline by SOC and PT
- Description of significant medical/surgical history by SOC and PT
- Number of patients with concomitant pathology by SOC and PT
- Description of concomitant pathologies by SOC and PT
- Number of patients with treated concomitant pathology by SOC and PT
- Description of treated concomitant pathologies by SOC and PT

ARTIALIS S.A., 11 Avenue de l’Hôpital, Sart-Tilman, tour GIGA, 4000 LIEGE – Belgium

Tel : 0032(0)4 2427706 – Fax : 0032(0)4 2427728

	ALL (N=XXX)	Group A (N=XXX)	Group B (N=XXX)	Group C (N=XXX)
Patients having significant medical/surgical history continuing at baseline	XX	XX	XX	XX
SOC - PT	XX (X%)	XX (X%)	XX (X%)	XX (X%)
SOC - PT	XX (X%)	XX (X%)	XX (X%)	XX (X%)
SOC - PT	XX (X%)	XX (X%)	XX (X%)	XX (X%)
Patients having significant medical/surgical history not continuing at baseline	XX	XX	XX	XX
SOC - PT	XX (X%)	XX (X%)	XX (X%)	XX (X%)
SOC - PT	XX (X%)	XX (X%)	XX (X%)	XX (X%)
SOC - PT	XX (X%)	XX (X%)	XX (X%)	XX (X%)

Table X: Number of patients with at least one significant medical/surgical history continuing/not continuing at baseline by SOC and PT

	ALL (N=XXX)	Group A (N=XXX)	Group B (N=XXX)	Group C (N=XXX)
Significant medical/surgery history continuing at baseline	XX	XX	XX	XX
SOC - PT	XX (X%)	XX (X%)	XX (X%)	XX (X%)
SOC - PT	XX (X%)	XX (X%)	XX (X%)	XX (X%)
SOC - PT	XX (X%)	XX (X%)	XX (X%)	XX (X%)
Significant medical/surgery history not continuing at baseline	XX	XX	XX	XX
SOC - PT	XX (X%)	XX (X%)	XX (X%)	XX (X%)
SOC - PT	XX (X%)	XX (X%)	XX (X%)	XX (X%)
SOC - PT	XX (X%)	XX (X%)	XX (X%)	XX (X%)

Table X: Description of significant medical/surgical history by SOC and PT

Knee OA history, treatment history and pain assessment

Knee OA characteristics will be summarized for both FAS and PP populations.

Knee OA history and pain assessment

Categorical variables will be summarized per group and globally using the frequency and percentage. Khi2, Fisher exact test or Kruskal-Wallis tests will be performed. Pairwise comparisons will be performed if significant:

- affected knee (left/right),

- K&L grade of the knee (0 to IV),
- presence of chondrocalcinosis
- other OA sites (contralateral knee, hip, hand, spine, unknown, other)

Continuous variables will be summarized per group and globally using the arithmetic mean, standard deviation, median, 1st and 3rd quartiles and range. ANOVA will be performed to evaluate differences between treatment groups. If significant, Tukey Post hoc tests will be performed between groups. Assessment of the normality of data will be performed with Shapiro-Wilk normality test when n≤50 and with Kolmogorov-smirnov when n >50. If not Normal the analyses will be conducted on rank:

- Mean knee pain of the last 24 hours (VAS)
- History of knee pain score in the last 3 months (VAS)
- Global assessment of disease activity (VAS)
- KOOS scores (global KOOS score and sub-scores)
- Duration of knee OA (based on the date of first symptoms)

		ALL (N=XXX)	Group A (N=XXX)	Group B (N=XXX)	Group C (N=XXX)	P-Value
**Knee OA history and pain assessment**						
Affected knee	Left	XXX/XXX (XX.X%)	XXX/XXX (XX.X%)	XXX/XXX (XX.X%)	XXX/XXX (XX.X%)	<0.001(S)†
	Right	XXX/XXX (XX.X%)	XXX/XXX (XX.X%)	XXX/XXX (XX.X%)	XXX/XXX (XX.X%)	
	Both	XXX/XXX (XX.X%)	XXX/XXX (XX.X%)	XXX/XXX (XX.X%)	XXX/XXX (XX.X%)	
KL grade	0	XXX/XXX (XX.X%)	XXX/XXX (XX.X%)	XXX/XXX (XX.X%)	XXX/XXX (XX.X%)	0.071(NS)*
	1	XXX/XXX (XX.X%)	XXX/XXX (XX.X%)	XXX/XXX (XX.X%)	XXX/XXX (XX.X%)	
	2	XXX/XXX (XX.X%)	XXX/XXX (XX.X%)	XXX/XXX (XX.X%)	XXX/XXX (XX.X%)	
	3	XXX/XXX (XX.X%)	XXX/XXX (XX.X%)	XXX/XXX (XX.X%)	XXX/XXX (XX.X%)	
	4	XXX/XXX (XX.X%)	XXX/XXX (XX.X%)	XXX/XXX (XX.X%)	XXX/XXX (XX.X%)	
Chondrocalcinosis	Yes	XXX/XXX (XX.X%)	XXX/XXX (XX.X%)	XXX/XXX (XX.X%)	XXX/XXX (XX.X%)	<0.001(S)†
	No	XXX/XXX (XX.X%)	XXX/XXX (XX.X%)	XXX/XXX (XX.X%)	XXX/XXX (XX.X%)	
Other OA sites	Contralateral knee	XXX/XXX (XX.X%)	XXX/XXX (XX.X%)	XXX/XXX (XX.X%)	XXX/XXX (XX.X%)	<0.001(S)†
	Hip	XXX/XXX (XX.X%)	XXX/XXX (XX.X%)	XXX/XXX (XX.X%)	XXX/XXX (XX.X%)	
	Hand	XXX/XXX (XX.X%)	XXX/XXX (XX.X%)	XXX/XXX (XX.X%)	XXX/XXX (XX.X%)	
	Spine	XXX/XXX (XX.X%)	XXX/XXX (XX.X%)	XXX/XXX (XX.X%)	XXX/XXX (XX.X%)	

	unkown	XXX/XXX (XX.X%)	XXX/XXX (XX.X%)	XXX/XXX (XX.X%)	XXX/XXX (XX.X%)	
	Other	XXX/XXX (XX.X%)	XXX/XXX (XX.X%)	XXX/XXX (XX.X%)	XXX/XXX (XX.X%)	
Mean knee pain of the last 24 hours (VAS)	N (MD)	XXX (XXX)	XXX (XXX)	XXX (XXX)	XXX (XXX)	0.005 (S)§
	Mean (SD)	XX.X (X.X)	XX.X (X.X)	XX.X (X.X)	XX.X (X.X)	
	Median [Q1-Q3]	XX.X [XX.X; XX.X]	XX.X [XX.X; XX.X]	XX.X [XX.X; XX.X]	XX.X [XX.X; XX.X]	
	Min - Max	XXX - XXX	XXX - XXX	XXX - XXX	XXX - XXX	
History of knee pain score in the last 3 months (VAS)	N (MD)	XXX (XXX)	XXX (XXX)	XXX (XXX)	XXX (XXX)	0.005 (S)§
	Mean (SD)	XX.X (X.X)	XX.X (X.X)	XX.X (X.X)	XX.X (X.X)	
	Median [Q1-Q3]	XX.X [XX.X; XX.X]	XX.X [XX.X; XX.X]	XX.X [XX.X; XX.X]	XX.X [XX.X; XX.X]	
	Min - Max	XXX - XXX	XXX - XXX	XXX - XXX	XXX - XXX	
Global assessment of disease activity (VAS)	N (MD)	XXX (XXX)	XXX (XXX)	XXX (XXX)	XXX (XXX)	0.005 (S)§
	Mean (SD)	XX.X (X.X)	XX.X (X.X)	XX.X (X.X)	XX.X (X.X)	
	Median [Q1-Q3]	XX.X [XX.X; XX.X]	XX.X [XX.X; XX.X]	XX.X [XX.X; XX.X]	XX.X [XX.X; XX.X]	
	Min - Max	XXX - XXX	XXX - XXX	XXX - XXX	XXX - XXX	
KOOS (pain sub-score)	N (MD)	XXX (XXX)	XXX (XXX)	XXX (XXX)	XXX (XXX)	0.005 (S)§
	Mean (SD)	XX.X (X.X)	XX.X (X.X)	XX.X (X.X)	XX.X (X.X)	
	Median [Q1-Q3]	XX.X [XX.X; XX.X]	XX.X [XX.X; XX.X]	XX.X [XX.X; XX.X]	XX.X [XX.X; XX.X]	
	Min - Max	XXX - XXX	XXX - XXX	XXX - XXX	XXX - XXX	
Duration of knee OA (based on the date of first symptoms)	N (MD)	XXX (XXX)	XXX (XXX)	XXX (XXX)	XXX (XXX)	0.005 (S)§
	Mean (SD)	XX.X (X.X)	XX.X (X.X)	XX.X (X.X)	XX.X (X.X)	
	Median [Q1-Q3]	XX.X [XX.X; XX.X]	XX.X [XX.X; XX.X]	XX.X [XX.X; XX.X]	XX.X [XX.X; XX.X]	
	Min - Max	XXX - XXX	XXX - XXX	XXX - XXX	XXX - XXX	

* Kruskal-Wallis test
† Chi-Square test
§ ANOVA test

Table 4 Knee OA history and Pain assessment. Baseline Conditions. PP population

If significant, Tukey Post hoc tests will be performed:

Comparison	Means difference	Adjusted P-value* (Significance)	Adjusted 95% CI
Group A vs. Group B	X.XXX	X.XXX (Not significant)	[X.XXX; X.XXX]
Group A vs. Group C	X.XXX	X.XXX **(Significant)**	[X.XXX; X.XXX]
Group B vs. Group C	X.XXX	X.XXX **(Significant)**	[X.XXX; X.XXX]

* Bilateral Tukey adjustment

Table 5 Knee OA history and Pain assessment: Mean knee pain of the last 24 hours (VAS). Baseline Conditions. Pairwise comparisons. PP population

Knee OA treatment history

Categorical variables will be summarized using the frequency and percentage. Khi2, Fisher exact or, for ordinal variables, Kruskall Wallis tests will be performed to assess differences between groups:

- Corticosteroids injection
- Hyaluronan injection
- Oral corticotherapy ≥5mg/day (Prednisone equivalent)

- Use of paracetamol for the knee pain in the last month
    - Will be categorized as number of days of uptake in the last month
    - Main dosage forms will be described by frequency (%)
- Use of oral NSAID drugs for the knee pain in the last month
    - Will be categorized as number of days of uptake in the last month
    - Main dosage forms will be described by frequency (%)

		ALL (N=XXX)	Group A (N=XXX)	Group B (N=XXX)	Group C (N=XXX)	P-Value
**Knee OA Treatment history**						
Corticosteroids injection	No	XXX/XXX (XX.X%)	XXX/XXX (XX.X%)	XXX/XXX (XX.X%)	XXX/XXX (XX.X%)	0.071(NS)*
	In the last 3 months	XXX/XXX (XX.X%)	XXX/XXX (XX.X%)	XXX/XXX (XX.X%)	XXX/XXX (XX.X%)	
	Between 3-6 months	XXX/XXX (XX.X%)	XXX/XXX (XX.X%)	XXX/XXX (XX.X%)	XXX/XXX (XX.X%)	
	> 6 months	XXX/XXX (XX.X%)	XXX/XXX (XX.X%)	XXX/XXX (XX.X%)	XXX/XXX (XX.X%)	

*Kruskal-Wallis test
† Chi-Square test

Table 6 Knee OA treatment history. Baseline Conditions. PP population

Biomarkers Baseline Data

Biomarkers (Coll2-1 and ultrasensitive CRP) baseline characteristics (Inclusion visit T0) of both PP and FAS populations will be summarized in a table using N, number of missing data (MD), arithmetic mean, standard deviation, median, 1st and 3rd quartile, and min-max. Comparisons will be evaluated using ANOVA with Tukey Post hoc test. Model p-values will be reported. Assessment of the normality of data will be performed with Shapiro-Wilk normality test when n≤50 and with Kolmogorov-smirnov when n >50. If not Normal the analyses will be conducted on rank.

		ALL (N=XXX)	Group A (N=XXX)	Group B (N=XXX)	Group C (N=XXX)	P-Value
**Biomarker baseline Data**						
Coll2-1	N (MD)	XXX (XXX)	XXX (XXX)	XXX (XXX)	XXX (XXX)	0.005 (S)§
	Mean (SD)	XX.X (X.X)	XX.X (X.X)	XX.X (X.X)	XX.X (X.X)	
	Median [Q1-Q3]	XX.X [XX.X; XX.X]	XX.X [XX.X; XX.X]	XX.X [XX.X; XX.X]	XX.X [XX.X; XX.X]	
	Min - Max	XXX - XXX	XXX - XXX	XXX - XXX	XXX - XXX	

		ALL (N=XXX)	Group A (N=XXX)	Group B (N=XXX)	Group C (N=XXX)	P-Value
Ultrasensitive CRP	N (MD)	XXX (XXX)	XXX (XXX)	XXX (XXX)	XXX (XXX)	0.005 (S)[§]
	Mean (SD)	XX.X (X.X)	XX.X (X.X)	XX.X (X.X)	XX.X (X.X)	
	Median [Q1-Q3]	XX.X [XX.X; XX.X]	XX.X [XX.X; XX.X]	XX.X [XX.X; XX.X]	XX.X [XX.X; XX.X]	
	Min - Max	XXX - XXX	XXX - XXX	XXX - XXX	XXX - XXX	

[§] ANOVA test

Table 7 Biomarkers Baseline Data: scoll2-1 and ultrasensitive CRP. PP population

If significant, Tukey Post hoc tests will be performed:

Comparison	Means difference	Adjusted P-value* (Significance)	Adjusted 95% CI
Group A vs. Group B	X.XXX	X.XXX (Not significant)	[X.XXX; X.XXX]
Group A vs. Group C	X.XXX	X.XXX **(Significant)**	[X.XXX; X.XXX]
Group B vs. Group C	X.XXX	X.XXX **(Significant)**	[X.XXX; X.XXX]

* Bilateral Tukey adjustment

Table 8 Biomarker Baseline Data: Coll2-1. Pairwise comparisons. PP population

Biomarkers (Coll2-1 and ultrasensitive CRP) at baseline will be graphically represented using nonparametric box-and-whiskers plots.

9.4. EFFICACY ANALYSES

All the efficacy analyses for primary and secondary variables and statistical analysis will be performed for both FAS and PP populations.

9.4.1. Target variables

Primary Target Variables

Biochemical target variable: The absolute value of biomarker **sColl2-1** in serum.

Clinical target variable: **Global assessment of disease activity** determined by a visual analogue scale (VAS): the VAS absolute value.
All primary target variables will be considered as continuous variables.

Secondary Target Variables

- The absolute value of biomarker **ultrasensitive CRP** in serum.
- **Mean knee pain** over the last 24 hours determined by a visual analogue scale (VAS)
- **Functional ability** determined by the Knee injury and Osteoarthritis Outcome Score (**KOOS**) index and its subscale scores (a self-administered questionnaire)
- Use of rescue treatments i.e. Paracetamol and oral NSAIDs during the study
- Satisfaction determined by a satisfaction scale
- Occurrence of Adverse events (AE), drop-outs and blood safety on patients with AE (Tolerance)
- Compliance determined by the pill count and curcumin blood level monitoring (compliance)

9.4.2. Methods of Efficacy analysis

All efficacy analyses for primary and secondary variables will be performed for both FAS and PP populations.

9.4.3. Efficacy Descriptive Analysis

Continuous Primary and Secondary Efficacy variables, namely biomarker Coll2-1 and global assessment of disease activity (VAS); ultrasensitive CRP, mean knee pain over the last 24 hours (VAS), global KOOS and its subscales, curcumin blood level (7 metabolites), will be summarized with descriptive statistics at each time point for each treatment group and globally (N, number of missing data (MD), arithmetic mean, standard deviation, median, 1st and 3rd quartile, and range).

		ALL (N=XXX)	Group A (N=XXX)	Group B (N=XXX)	Group C (N=XXX)
**Biomarker sColl2-1**					
T0	N (MD)	XXX (XXX)	XXX (XXX)	XXX (XXX)	XXX (XXX)
	Mean (SD)	XX.X (X.X)	XX.X (X.X)	XX.X (X.X)	XX.X (X.X)
	Median [Q1-Q3]	XX.X [XX.X; XX.X]	XX.X [XX.X; XX.X]	XX.X [XX.X; XX.X]	XX.X [XX.X; XX.X]
	Min - Max	XXX - XXX	XXX - XXX	XXX - XXX	XXX - XXX
T1	N (MD)	XXX (XXX)	XXX (XXX)	XXX (XXX)	XXX (XXX)
	Mean (SD)	XX.X (X.X)	XX.X (X.X)	XX.X (X.X)	XX.X (X.X)
	Median [Q1-Q3]	XX.X [XX.X; XX.X]	XX.X [XX.X; XX.X]	XX.X [XX.X; XX.X]	XX.X [XX.X; XX.X]
	Min - Max	XXX - XXX	XXX - XXX	XXX - XXX	XXX - XXX
T3	N (MD)	XXX (XXX)	XXX (XXX)	XXX (XXX)	XXX (XXX)
	Mean (SD)	XX.X (X.X)	XX.X (X.X)	XX.X (X.X)	XX.X (X.X)
	Median [Q1-Q3]	XX.X [XX.X; XX.X]	XX.X [XX.X; XX.X]	XX.X [XX.X; XX.X]	XX.X [XX.X; XX.X]
	Min - Max	XXX - XXX	XXX - XXX	XXX - XXX	XXX - XXX
T6	N (MD)	XXX (XXX)	XXX (XXX)	XXX (XXX)	XXX (XXX)
	Mean (SD)	XX.X (X.X)	XX.X (X.X)	XX.X (X.X)	XX.X (X.X)
	Median [Q1-Q3]	XX.X [XX.X; XX.X]	XX.X [XX.X; XX.X]	XX.X [XX.X; XX.X]	XX.X [XX.X; XX.X]
	Min - Max	XXX - XXX	XXX - XXX	XXX - XXX	XXX - XXX
Change T1	N (MD)	XXX (XXX)	XXX (XXX)	XXX (XXX)	XXX (XXX)
	Mean (SD)	XX.X (X.X)	XX.X (X.X)	XX.X (X.X)	XX.X (X.X)
	Median [Q1-Q3]	XX.X [XX.X; XX.X]	XX.X [XX.X; XX.X]	XX.X [XX.X; XX.X]	XX.X [XX.X; XX.X]
	Min - Max	XXX - XXX	XXX - XXX	XXX - XXX	XXX - XXX
Change T3	N (MD)	XXX (XXX)	XXX (XXX)	XXX (XXX)	XXX (XXX)
	Mean (SD)	XX.X (X.X)	XX.X (X.X)	XX.X (X.X)	XX.X (X.X)
	Median [Q1-Q3]	XX.X [XX.X; XX.X]	XX.X [XX.X; XX.X]	XX.X [XX.X; XX.X]	XX.X [XX.X; XX.X]
	Min - Max	XXX - XXX	XXX - XXX	XXX - XXX	XXX - XXX
Change T6	N (MD)	XXX (XXX)	XXX (XXX)	XXX (XXX)	XXX (XXX)
	Mean (SD)	XX.X (X.X)	XX.X (X.X)	XX.X (X.X)	XX.X (X.X)
	Median [Q1-Q3]	XX.X [XX.X; XX.X]	XX.X [XX.X; XX.X]	XX.X [XX.X; XX.X]	XX.X [XX.X; XX.X]

	ALL (N=XXX)	Group A (N=XXX)	Group B (N=XXX)	Group C (N=XXX)
Min - Max	XXX - XXX	XXX - XXX	XXX - XXX	XXX - XXX

Table 9 Efficacy descriptive Analysis: Biomarker sColl2-1. PP Population

Categorical and ordinal data, namely Patient' satisfaction with treatment, pill count and use of paracetamol and oral NSAIDs for knee pain in the last month will be summarized using percentages and frequency, per group and globally at each time point.

		ALL (N=XXX)	Group A (N=XXX)	Group B (N=XXX)	Group C (N=XXX)
**Categorial** **Secondary efficacy variables**					
Oral NSAIDs for knee pain in the last month (T0)	Yes	XXX/XXX (XX.X%)	XXX/XXX (XX.X%)	XXX/XXX (XX.X%)	XXX/XXX (XX.X%)
	No	XXX/XXX (XX.X%)	XXX/XXX (XX.X%)	XXX/XXX (XX.X%)	XXX/XXX (XX.X%)
Oral NSAIDs for knee pain in the last month (T0)	Less than 7 days	XXX/XXX (XX.X%)	XXX/XXX (XX.X%)	XXX/XXX (XX.X%)	XXX/XXX (XX.X%)
	7 to 14 days	XXX/XXX (XX.X%)	XXX/XXX (XX.X%)	XXX/XXX (XX.X%)	XXX/XXX (XX.X%)
	15 to 21 days	XXX/XXX (XX.X%)	XXX/XXX (XX.X%)	XXX/XXX (XX.X%)	XXX/XXX (XX.X%)
	More than 21 days	XXX/XXX (XX.X%)	XXX/XXX (XX.X%)	XXX/XXX (XX.X%)	XXX/XXX (XX.X%)
	everyday	XXX/XXX (XX.X%)	XXX/XXX (XX.X%)	XXX/XXX (XX.X%)	XXX/XXX (XX.X%)
Oral NSAIDs for knee pain in the last month (T1)	Yes	XXX/XXX (XX.X%)	XXX/XXX (XX.X%)	XXX/XXX (XX.X%)	XXX/XXX (XX.X%)
	No	XXX/XXX (XX.X%)	XXX/XXX (XX.X%)	XXX/XXX (XX.X%)	XXX/XXX (XX.X%)
Oral NSAIDs for knee pain in the last month (T1)	Less than 7 days	XXX/XXX (XX.X%)	XXX/XXX (XX.X%)	XXX/XXX (XX.X%)	XXX/XXX (XX.X%)
	7 to 14 days	XXX/XXX (XX.X%)	XXX/XXX (XX.X%)	XXX/XXX (XX.X%)	XXX/XXX (XX.X%)
	15 to 21 days	XXX/XXX (XX.X%)	XXX/XXX (XX.X%)	XXX/XXX (XX.X%)	XXX/XXX (XX.X%)
	More than 21 days	XXX/XXX (XX.X%)	XXX/XXX (XX.X%)	XXX/XXX (XX.X%)	XXX/XXX (XX.X%)
	everyday	XXX/XXX (XX.X%)	XXX/XXX (XX.X%)	XXX/XXX (XX.X%)	XXX/XXX (XX.X%)
Oral NSAIDs for knee pain in the last month (T3)	Yes	XXX/XXX (XX.X%)	XXX/XXX (XX.X%)	XXX/XXX (XX.X%)	XXX/XXX (XX.X%)
	No	XXX/XXX (XX.X%)	XXX/XXX (XX.X%)	XXX/XXX (XX.X%)	XXX/XXX (XX.X%)

Oral NSAIDs for knee pain in the last month (T3)	Less than 7 days	XXX/XXX (XX.X%)	XXX/XXX (XX.X%)	XXX/XXX (XX.X%)	XXX/XXX (XX.X%)
	7 to 14 days	XXX/XXX (XX.X%)	XXX/XXX (XX.X%)	XXX/XXX (XX.X%)	XXX/XXX (XX.X%)
	15 to 21 days	XXX/XXX (XX.X%)	XXX/XXX (XX.X%)	XXX/XXX (XX.X%)	XXX/XXX (XX.X%)
	More than 21 days	XXX/XXX (XX.X%)	XXX/XXX (XX.X%)	XXX/XXX (XX.X%)	XXX/XXX (XX.X%)
	everyday	XXX/XXX (XX.X%)	XXX/XXX (XX.X%)	XXX/XXX (XX.X%)	XXX/XXX (XX.X%)
Oral NSAIDs for knee pain in the last month (T6)	Yes	XXX/XXX (XX.X%)	XXX/XXX (XX.X%)	XXX/XXX (XX.X%)	XXX/XXX (XX.X%)
	No	XXX/XXX (XX.X%)	XXX/XXX (XX.X%)	XXX/XXX (XX.X%)	XXX/XXX (XX.X%)
Oral NSAIDs for knee pain in the last month (T6)	Less than 7 days	XXX/XXX (XX.X%)	XXX/XXX (XX.X%)	XXX/XXX (XX.X%)	XXX/XXX (XX.X%)
	7 to 14 days	XXX/XXX (XX.X%)	XXX/XXX (XX.X%)	XXX/XXX (XX.X%)	XXX/XXX (XX.X%)
	15 to 21 days	XXX/XXX (XX.X%)	XXX/XXX (XX.X%)	XXX/XXX (XX.X%)	XXX/XXX (XX.X%)
	More than 21 days	XXX/XXX (XX.X%)	XXX/XXX (XX.X%)	XXX/XXX (XX.X%)	XXX/XXX (XX.X%)
	everyday	XXX/XXX (XX.X%)	XXX/XXX (XX.X%)	XXX/XXX (XX.X%)	XXX/XXX (XX.X%)

Table 10 Efficacy descriptive Analysis: Use of oral NSAIDS for knee pain in the last month. PP Population

Figures

Continuous efficacy variables (mean±SD) will be graphically displayed per treatment in function of time (concentration-time courses).

KOOS profiles

A plot representing mean KOOS score of the different time points versus KOOS subscales will be displayed for each treatment group (3 plots), as recommended in www.koos.nu:

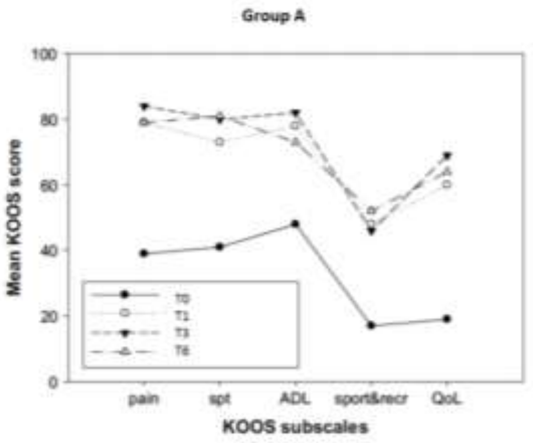

A plot representing mean KOOS score of the different groups versus KOOS subscales will be displayed for each timepoint (4 plots).

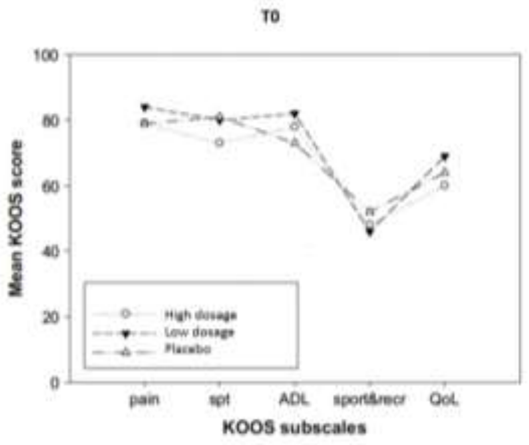

9.4.4. Treatment response - Primary Efficacy Variables

Primary target variables, namely Coll2-1 and VAS of global activity will be analyzed using a mixed model (repeated measures ANCOVA model) followed by post-hoc adjustments test. The response variable will be regressed on time (T0 and T3), treatment group (Group A, B and C) and their interaction:

$$Y = \alpha + \beta\ Treatment + \beta\ Time + \beta\ Treatment * Time + \varepsilon$$

An autoregressive variance-covariance matrix of this model will also consider the repeated structure of the measurements within each subject. Age and gender as between-subjects factors can be added to the model if they are significant. P-values for treatment effect, time effect and the effect of their interaction will be reported. In order to consider the two co-primaries criteria, the alpha level is set to 0.025.

Post-hoc pairwise comparisons will be performed.

If the interaction term is significant the post-hoc comparisons will be:

- between each treated group versus each of the two other groups within each timepoint, with bilateral Tukey adjustment test.
- between T0 and T3 within each group.

If the interaction term and the time terms are NOT significant while treatment is significant the post-hoc comparisons will be:

- between each treated group versus each of the two other groups globally over time, with bilateral Tukey adjustment test.

If the interaction term is NOT significant while the main term is significant and the time is also significant the post-hoc comparisons will be:

- between each treated group versus each of the two other groups globally over time, with bilateral Tukey adjustment test.
- between the time point globally over treatments.

Assessment of the normality of data will be performed with Shapiro-Wilk normality test when n≤50 and with Kolmogorov-smirnov when n >50. If not Normal the analyses will be conducted on rank.

The following table will be made:

Effect	P-value (Significance)
Treatment	X.XXX (Not significant)
Time	X.XXX **(Significant)**
Treatment*Time	X.XXX **(Significant)**

Table 11 Treatment responses: Biomarker sColl2-1, ANOVA model. PP Population

If found significant and <u>with interaction</u>, the following tables will be made:

<u>Between-groups comparisons</u>

Time	Comparison	Means difference	Adjusted P-value* (Significance)	Adjusted 97.5% CI
T0	Group A vs. Group B	X.XXX	X.XXX (Not significant)	[X.XXX; X.XXX]
	Group A vs. Group C	X.XXX	X.XXX **(Significant)**	[X.XXX; X.XXX]
	Group B vs. Group C	X.XXX	X.XXX **(Significant)**	[X.XXX; X.XXX]
T3	Group A vs. Group B	X.XXX	X.XXX (Not significant)	[X.XXX; X.XXX]
	Group A vs. Group C	X.XXX	X.XXX (Significant)	[X.XXX; X.XXX]
	Group B vs. Group C	X.XXX	X.XXX (Significant)	[X.XXX; X.XXX]

* Bilateral Tukey adjustment

Table 12 Treatment responses: Biomarker sColl2-1, RM-ANOVA model with Post Hoc Tukey adjustment. Between-groups comparisons. PP Population

<u>Within-groups comparisons</u>

Group	Comparison	Means difference	P-value (Significance)	Adjusted 97.5% CI
Group A	T3 vs T0	X.XXX	X.XXX **(Significant)**	[X.XXX; X.XXX]
Group B	T3 vs T0	X.XXX	X.XXX (Significant)	[X.XXX; X.XXX]
Group C	T3 vs T0	X.XXX	X.XXX (Significant)	[X.XXX; X.XXX]

Group	Comparison	Means difference	P-value (Significance)	Adjusted 97.5% CI

Table 13 Treatment responses: Biomarker sColl2-1, RM-ANOVA model. Within-groups comparisons. PP Population

If found significant <u>without interaction</u>, treatment and time effect will be reported globally:

If treatment effect is significant, it is analyzed globally (all timepoints)

<u>**treatment effect**</u>

Comparison	Means difference	Adjusted P-value* (Significance)	Adjusted 97.5% CI
Group A vs. Group B	X.XXX	X.XXX (Not significant)	[X.XXX; X.XXX]
Group A vs. Group C	X.XXX	X.XXX **(Significant)**	[X.XXX; X.XXX]
Group B vs. Group C	X.XXX	X.XXX **(Significant)**	[X.XXX; X.XXX]

* Bilateral Tukey adjustment

Table 14 Treatment responses: Biomarker sColl2-1, RM-ANOVA model with Post Hoc Tukey adjustment. Treatment groups comparisons. PP Population

If time effect is significant, it is analyzed globally (all groups)

<u>**Time effect**</u>

Comparison	Means difference	P-value (Significance)	Adjusted 97.5% CI
T3 vs T0	X.XXX	X.XXX (Not significant)	[X.XXX; X.XXX]

Table 15 Treatment responses: Biomarker sColl2-1, RM-ANOVA model. Time point comparisons. PP Population

The same analysis will be repeated using multiple imputation of missing data as sensitive analysis only for the PP population.

9.4.5. Treatment Response - Secondary Efficacy Variables

Continuous Variables

Continuous secondary target variables, namely:

- serum levels of sColl2-1
- serum levels of ultrasensitive CRP
- Mean knee pain over the last 24 hours using a visual analogue scale (VAS)
- Global assessment of disease activity using a VAS
- Knee injury and Osteoarthritis Outcome Score (KOOS) index and its subscale scores
- Curcumin blood level (curcumin, tetrahydrocurcumin, demethylcurcumin, didemethylcurcumin, hexahydrocurcumin, demethoxycurcumin and bisdemethoxycurcumin)

will be analyzed using a using a mixed model (repeated measures ANCOVA model) followed by post-hoc adjustments test.

The response variable will me regressed on time (T0, T1, T3, T6), treatment group (Group A, B and C) and their interaction:

$$Y = \alpha + \beta\, Treatment + \beta\, Time + \beta\, Treatment * Time + \varepsilon$$

An autoregressive variance-covariance matrix of this model will also consider the repeated structure of the measurements within each subject. Age and gender as between-subjects factors can be added to the model if they are significant. P-values for treatment effect, time effect and the effect of their interaction will be reported. Alpha level is 5%.

Post-hoc pairwise comparisons will be performed.

If the interaction term is significant the post-hoc comparisons will be:

- between each treated group versus each of the two other groups within each timepoint, with bilateral Tukey adjustment test.
- between each follow-up time point (T1, T3, T6) versus baseline (T0) within each group , with bilateral Dunett adjustment test.

If the interaction term is NOT significant and the treatment effect is significant but the time effect is not, the post-hoc comparisons will be:

- between each treated group versus each of the two other groups globally over time, with bilateral Tukey adjustment test.

If the interaction term is NOT significant and both, the treatment effect and the time effect are significant, the post-hoc comparisons will be:

- between each treated group versus each of the two other groups globally over time, with bilateral Tukey adjustment test.
- between each follow-up time point (T1, T3, T6) versus baseline (T0) globally over treatments, with bilateral Dunett adjustment test.

If assumptions for parametric tests are not verified, non-parametric Friedman's test with Bonferroni correction for multiple testing will be used.

Assessment of the normality of data will be performed with Shapiro-Wilk normality test when n≤50 and with Kolmogorov-smirnov when n >50. If not Normal the analyses will be conducted on rank.

The following table will be made:

Effect	P-value (Significance)
Treatment	X.XXX (Not significant)
Time	X.XXX **(Significant)**
Treatment*Time	X.XXX **(Significant)**

Table 16 Treatment responses: Biomarker sColl2-1, Repeated Measures ANOVA model. PP Population

If found significant and <u>with interaction</u>, the following tables will be made:

<u>Between-groups comparisons</u>

Time	Comparison	Means difference	Adjusted P-value* (Significance)	Adjusted 95% CI
T0	Group A vs. Group B	X.XXX	X.XXX (Not significant)	[X.XXX; X.XXX]
	Group A vs. Group C	X.XXX	X.XXX **(Significant)**	[X.XXX; X.XXX]
	Group B vs. Group C	X.XXX	X.XXX **(Significant)**	[X.XXX; X.XXX]
T1	Group A vs. Group B	X.XXX	X.XXX (Not significant)	[X.XXX; X.XXX]
	Group A vs. Group C	X.XXX	X.XXX **(Significant)**	[X.XXX; X.XXX]
	Group B vs. Group C	X.XXX	X.XXX **(Significant)**	[X.XXX; X.XXX]
T3	Group A vs. Group B	X.XXX	X.XXX (Not significant)	[X.XXX; X.XXX]
	Group A vs. Group C	X.XXX	X.XXX (Significant)	[X.XXX; X.XXX]
	Group B vs. Group C	X.XXX	X.XXX (Significant)	[X.XXX; X.XXX]
T6	Group A vs. Group B	X.XXX	X.XXX (Not significant)	[X.XXX; X.XXX]
	Group A vs. Group C	X.XXX	X.XXX (Significant)	[X.XXX; X.XXX]
	Group B vs. Group C	X.XXX	X.XXX (Significant)	[X.XXX; X.XXX]

* Bilateral Tukey adjustment

Table 17 Treatment responses: Biomarker sColl2-1, RM-ANOVA model with Post Hoc Tukey adjustment. Between-groups comparisons. PP Population

<u>Within-groups comparisons</u>

Group	Comparison	Means difference	Adjusted P-value* (Significance)	Adjusted 95% CI
Group A	T1 vs T0	X.XXX	X.XXX (Not significant)	[X.XXX; X.XXX]
	T3 vs T0	X.XXX	X.XXX **(Significant)**	[X.XXX; X.XXX]
	T6 vs T0	X.XXX	X.XXX **(Significant)**	[X.XXX; X.XXX]
Group B	T1 vs T0	X.XXX	X.XXX (Not significant)	[X.XXX; X.XXX]
	T3 vs T0	X.XXX	X.XXX (Significant)	[X.XXX; X.XXX]
	T6 vs T0	X.XXX	X.XXX (Significant)	[X.XXX; X.XXX]
Group C	T1 vs T0	X.XXX	X.XXX (Not significant)	[X.XXX; X.XXX]
	T3 vs T0	X.XXX	X.XXX (Significant)	[X.XXX; X.XXX]
	T6 vs T0	X.XXX	X.XXX (Significant)	[X.XXX; X.XXX]

Group	Comparison	Means difference	Adjusted P-value* (Significance)	Adjusted 95% CI

*Bilateral Dunnett Adjustment

Table 18 Treatment responses: Biomarker sColl2-1, RM-ANOVA model with Post Hoc Dunnet adjustment. Within-groups comparisons. PP Population

If found significant <u>without interaction</u>, treatment and time effect will be reported globally:
If treatment effect is significant, it is analyzed globally (all timepoints)

<u>Treatment effect</u>

Comparison	Means difference	Adjusted P-value* (Significance)	Adjusted 95% CI
Group A vs. Group B	X.XXX	X.XXX (Not significant)	[X.XXX; X.XXX]
Group A vs. Group C	X.XXX	X.XXX **(Significant)**	[X.XXX; X.XXX]
Group B vs. Group C	X.XXX	X.XXX **(Significant)**	[X.XXX; X.XXX]

* Bilateral Tukey adjustment

Table 19 Treatment responses: Biomarker sColl2-1, RM-ANOVA model with Post Hoc Tukey adjustment. Treatment groups comparisons. PP Population

If time effect is significant, it is analyzed globally (all groups)

<u>Time effect</u>

Comparison	Means difference	Adjusted P-value* (Significance)	Adjusted 95% CI
T1 vs T0	X.XXX	X.XXX (Not significant)	[X.XXX; X.XXX]
T3 vs T0	X.XXX	X.XXX (Not significant)	[X.XXX; X.XXX]
T6 vs T0	X.XXX	X.XXX **(Significant)**	[X.XXX; X.XXX]
…	X.XXX	X.XXX **(Significant)**	[X.XXX; X.XXX]

*Bilateral Dunnett Adjustment

Table 20 Treatment responses: Biomarker sColl2-1, RM-ANOVA model with Post Hoc Dunnet adjustment. Time point comparisons. PP Population

Categorical Variables

Categorical data of secondary efficacy variables will be presented as frequency counts and percentage of subjects within each category and globally. Khi2 or, for ordinal variables, Kruskall Wallis tests will be performed. If found significant, Dunn's nonparametric comparison post hoc testing will be performed.

<u>Use of rescue treatments (i.e. paracetamol and oral NSAIDs)</u> for the knee pain in the month prior to each visit (T0, T1, T3 and T6) will be categorized as yes/no and number of days of uptake in the last month. Khi2 test will be performed on the yes/no categorization while the Kruskall Wallis tests will be performed on the number of days.

Satisfaction with the investigational product between each visit (T0 to T1, T1 to T3 and T3 to T6) will be tabulated and analyzed with Kruskall Wallis test.

Compliance will be evaluated at each follow up visit by the Pill Count method : evaluated in % of the estimated versus theoretical consumption for the three groups. Kruskall Wallis tests will be performed.

		ALL (N=XXX)	Group A (N=XXX)	Group B (N=XXX)	Group C (N=XXX)	P-Value
**Categorial Secondary efficacy variables**						
Oral NSAIDs for knee pain in the last month (T0)	Yes	XXX/XXX (XX.X%)	XXX/XXX (XX.X%)	XXX/XXX (XX.X%)	XXX/XXX (XX.X%)	<0.001(S)†
	No	XXX/XXX (XX.X%)	XXX/XXX (XX.X%)	XXX/XXX (XX.X%)	XXX/XXX (XX.X%)	
Oral NSAIDs for knee pain in the last month (T1)	Yes	XXX/XXX (XX.X%)	XXX/XXX (XX.X%)	XXX/XXX (XX.X%)	XXX/XXX (XX.X%)	<0.001(S)†
	No	XXX/XXX (XX.X%)	XXX/XXX (XX.X%)	XXX/XXX (XX.X%)	XXX/XXX (XX.X%)	
Oral NSAIDs for knee pain in the last month (T3)	Yes	XXX/XXX (XX.X%)	XXX/XXX (XX.X%)	XXX/XXX (XX.X%)	XXX/XXX (XX.X%)	<0.001(S)†
	No	XXX/XXX (XX.X%)	XXX/XXX (XX.X%)	XXX/XXX (XX.X%)	XXX/XXX (XX.X%)	
Oral NSAIDs for knee pain in the last month (T6)	Yes	XXX/XXX (XX.X%)	XXX/XXX (XX.X%)	XXX/XXX (XX.X%)	XXX/XXX (XX.X%)	<0.001(S)†
	No	XXX/XXX (XX.X%)	XXX/XXX (XX.X%)	XXX/XXX (XX.X%)	XXX/XXX (XX.X%)	

† Chi-Square test

Table 21 Treatment responses: Use of oral NSAIDs for the knee pain in the last month (yes/no), khi square test. PP Population

	ALL (N=XXX)	Group A (N=XXX)	Group B (N=XXX)	Group C (N=XXX)	P-Value
**Categorial Secondary efficacy variables**					

		ALL (N=XXX)	Group A (N=XXX)	Group B (N=XXX)	Group C (N=XXX)	P-Value
Oral NSAIDs for knee pain in the last month (T0)	Less than 7 days	XXX/XXX (XX.X%)	XXX/XXX (XX.X%)	XXX/XXX (XX.X%)	XXX/XXX (XX.X%)	<0.001(S)*
	7 to 14 days	XXX/XXX (XX.X%)	XXX/XXX (XX.X%)	XXX/XXX (XX.X%)	XXX/XXX (XX.X%)	
	15 to 21 days	XXX/XXX (XX.X%)	XXX/XXX (XX.X%)	XXX/XXX (XX.X%)	XXX/XXX (XX.X%)	
	More than 21 days	XXX/XXX (XX.X%)	XXX/XXX (XX.X%)	XXX/XXX (XX.X%)	XXX/XXX (XX.X%)	
	everyday	XXX/XXX (XX.X%)	XXX/XXX (XX.X%)	XXX/XXX (XX.X%)	XXX/XXX (XX.X%)	
Oral NSAIDs for knee pain in the last month (T1)	Less than 7 days	XXX/XXX (XX.X%)	XXX/XXX (XX.X%)	XXX/XXX (XX.X%)	XXX/XXX (XX.X%)	0.005 (S)*
	7 to 14 days	XXX/XXX (XX.X%)	XXX/XXX (XX.X%)	XXX/XXX (XX.X%)	XXX/XXX (XX.X%)	
	15 to 21 days	XXX/XXX (XX.X%)	XXX/XXX (XX.X%)	XXX/XXX (XX.X%)	XXX/XXX (XX.X%)	
	More than 21 days	XXX/XXX (XX.X%)	XXX/XXX (XX.X%)	XXX/XXX (XX.X%)	XXX/XXX (XX.X%)	
	everyday	XXX/XXX (XX.X%)	XXX/XXX (XX.X%)	XXX/XXX (XX.X%)	XXX/XXX (XX.X%)	

* Kruskal-Wallis test

Table 22 Treatment responses: Use of oral NSAIDs for the knee pain in the last month (number of days of uptake), Kruskal-Wallis test. PP Population

9.5. SAFETY AND TOLERABILITY ANALYSES

Safety and tolerability of Flexofytol will be evaluated descriptively for each treatment group (for categorical data: N, %, for quantitative data: N, arithmetic mean, standard deviation, median, minimum and maximum), based on the SP population with respect to the following parameters collected for each subject:

- Adverse Events
  - AEs and SAEs
  - AEs leading to withdrawal
  - AEs linked to the study product
  - Any Deaths
- Pregnancies
- Concomitant Medications

Blood safety (hepatic and renal function) will be collected and analyzed for subjects with AE linked to the product only.

9.5.1. Adverse events

All adverse events

Summaries of diagnosis, severity, relationship to study drug, outcome and duration of individual AEs will be prepared. Adverse Event recorded during the study will be coded using MedDRA version 18.0. Medication will be coded using the ATC/DDD Index 2015 recommended by the WHO.

Only AEs that appear at or after the beginning of study drug administration will be included.

Patient	Treatment Group	Diagnosis / Symptoms	Severity	Relationship to the product	Action taken	Outcome	Time to onset	Duration	Serious AE

Table 23 Listing of AE (SP)

Summaries of number of AE per patient and percentage of patients with AE, AE related to the drug and SAE will be prepared. Treated groups will be compared with a Khi2 test (Fischer exact test in case one or more cells has a frequency of five or less) for the categorical variables and, for ordinal variables, with a Kruskall Wallis tests.

Number of AE per patient	Treatment						Total		Khi² Test
	High dosage		Low dosage		Placebo				
	N	%	N	%	N	%	N	%	
0									Khi² :<br>p-value :
1									Khi² :<br>p-value :
2									Khi² :<br>p-value :
3									Khi² :<br>p-value :
Total		100		100		100		100	

Table 24 Number of AE per patient (SP)

Kruskall Wallis : p-value : Signification :

ARTIALIS S.A., 11 Avenue de l'Hôpital, Sart-Tilman, tour GIGA, 4000 LIEGE – Belgium

Tel : 0032(0)4 2427706 – Fax : 0032(0)4 2427728

Number of AE per patient	Treatment						Total		Khi² Test
	High dosage		Low dosage		Placebo				
	N	%	N	%	N	%	N	%	
% patients with at least 1 AE								100	Khi² :<br>p-value :
% patients with at least 1 AE related to IP								100	Khi² :<br>p-value :
% patients with at least 1 SAE								100	Khi² :<br>p-value :

Table 25 Number of AE per patient (SP)

Kruskall Wallis : p-value : Signification :

A listing of adverse events, System Organ Class (SOC), Preferred Term, severity, relation to the treatment, action and evolution will be prepared for each treatment group.

SOC	PT	Severity			Relationship to the Product		Action taken					Outcome			
		mild	moderate	severe	yes	No	None	Withdraw	Treatment	Medical consultation	Hospitalization	Recovered	Recovered with sequelea	Continuing	Death

Table 26 Listing of AE (SOC and PT), severity, relationship to product, action taken and outcome. Treatment group XX (SP)

The onset of events relative to study drug administration will be presented graphically if appropriate.

Adverse events linked to the product and/or leading to withdrawal

A summary of incidence rates (frequencies and percentages) of AEs linked to the product and/or leading to withdrawal from the study will be prepared for the Safety Population.

	Treatment						Total		Khi² Test
	High dosage		Low dosage		Placebo				
	N	%	N	%	N	%	N	%	
Number of AEs leading to withdrawal								100	Khi² :<br>p-value :
Number of AEs linked to the product								100	Khi² :<br>p-value :
Number of AEs linked to the product and leading to withdrawal								100	Khi² :<br>p-value :

A data listing of AEs leading to withdrawal will also be provided, displaying details of the event(s) captured on the CRF.

Serious adverse events

Serious AE reconciliation will be performed by the pharmacovigilance responsible person at Sponsor.

A summary of incidence rates (frequencies and percentages) of SAEs, System Organ Class, Preferred Term, severity, relation to the treatment, action and evolution will be prepared for the Safety Population.

Deaths

If any subject dies during the study, relevant information will be supplied in a data listing, and appropriate SAE narratives.

9.5.2. Pregnancies

Pregnancy data will be shown in a data listing. No special analysis will be performed on the pregnancy data. Subjects are to be discontinued from the study if they become pregnant.

9.5.3. Clinical Laboratory Evaluations

Laboratory evaluations include urea, creatinine, uric acid, total bilirubin, conjugated bilirubin, GGT, TGO and TGP.

Descriptive summaries (arithmetic mean, SD, median, minimum, and maximum) will be presented for blood safety values for subjects with adverse events linked to the product.

The number of subjects with clinical laboratory values below, within, or above normal ranges will be tabulated. Blood safety values will be compared between treatment groups.

Laboratory values of each subject at all available timepoints will also be presented.

Laboratory values – Renal function

Urea (mg/dL)

Patient	Treatment Group	T0	T1	T3	T6

Creatinin (mg/dL)

Patient	Treatment Group	T0	T1	T3	T6	Ref value

Uric acid (mg/dL)

Patient	Treatment Group	T0	T1	T3	T6	Ref value
						<6
						<6
						<6
						<6

Laboratory values – Liver function

Total Bilirubin (mg/dL)

Patient	Treatment Group	T0	T1	T3	T6	Ref value
						<1.20
						<1.20
						<1.20
						<1.20

Conjugated Bilirubin (mg/dL)

Patient	Treatment Group	T0	T1	T3	T6	Ref value
						<0.39
						<0.39
						<0.39
						<0.39

GGT (U/L)

Patient	Treatment Group	T0	T1	T3	T6

TGO (ASAT) (U/L)

Patient	Treatment Group	T0	T1	T3	T6

TGP (ALAT) (U/L)

Patient	Treatment Group	T0	T1	T3	T6

9.6. PATIENTS LISTING

Additional to the tables and listings given above the following listings will be reported by patients and if it is relevant, by study period:

- Discontinued patients
- Protocol deviations
- Patients excluded from the efficacy analysis
- Demographic data
- Prior and Concomitant Medications
- Medical history and concomitant pathologies
- Knee OA history
- Knee OA pain assessment
- Knee OA treatment history
- Biomarkers Baseline Data
- Pill count and curcumin blood level

- Coll2-1
- Global assessment of disease activity (VAS)
- Ultrasensitive CRP
- Mean knee pain over the last 24 hours (VAS)
- Global KOOS and its subscales
- Rescue treatments for knee pain in the last month
- Patient' satisfaction with treatment

9.7. COMPLEMENTARY EXPLORATORY ANALYSES

Exploratory analyses will be performed as a second stage on biomarkers and clinical data.

9.7.1. General Aspects

Distribution of the quantitative data will be investigated. Non-parametric tests will be used in case of non-normality of the distributions. If necessary, for multivariate approaches, log-transformation will be applied. The presence of missing data will be investigated and appropriate approaches considered. All tests will be two-sided and results will be considered significant at the 5% critical level. Statistical calculations will be carried out using the SAS (version 9.4 for Windows) package.

9.7.2. Statistical analysis – Specific aspects

Correlation

In both groups treated with Flexofytol, correlations between the 7 curcumin metabolites and both principal criteria (Coll2-1 and VAS global activity) will be calculated at each time point. The same scheme will be applied to the evolution of both principal criteria measured at 3 months. In order to account for the presence of missing, a sensitivity analysis based on multiple imputation process will be applied.

Evolution in sub-groups

The evolution across the four time points of the Coll2-1 and of the VAS global activity will be compared between the three treatment groups in different sub-samples. In that purpose, a mixed model will be fitted to the data to test for differences between the treatment groups. The covariates included in the model will be the time and the interaction with the treatment indicator. This statistical method permits the comparison of response curves between treatments while accounting for repeated data within patients. The model considered in this part allows accounting for missing data.

Four sub-sample analyses will be considered:

1. Pain at baseline: three sub-groups will be constructed based on the observed tertile values.

ARTIALIS S.A., 11 Avenue de l'Hôpital, Sart-Tilman, tour GIGA, 4000 LIEGE – Belgium

Tel : 0032(0)4 2427706 – Fax : 0032(0)4 2427728

2. Kellgren and Lawrence grade at baseline: two sub-groups will be constructed based on the observed scores (2 vs 3).

3. CRP level measured at baseline: three sub-groups will be constructed based on the observed tertile values.

4. Coll2-1 level measured at baseline: three sub-groups will be constructed based on the observed tertile values.

Time Integrated concentrations

The method of Time integrated concentration will be applied in each group. This method will be applied to Coll2-1 as well as to the VAS global activity.

10. REPORTING CONVENTIONS

The following reporting conventions will be adopted for the SAP. These conventions will enhance the review process and help to standardize presentation with common notations.

10.1. General Reporting Conventions

- Legends will be used for all figures with more than one variable or item displayed.
- Only standard keyboard characters should be used in tables and data listings. Special characters, such as non-printable control characters, printer specific, or font specific characters, will not be used on a table, figure, or data listing. Hexadecimal character representations are allowed (*e.g.*, μ, α, β).
- Missing values for both numeric and character variables will be presented as "MV" in a table or data listing.
- All date values will be presented as DD.MMM.YYYY (*e.g.*, 29.AUG.2001) format. A four-digit year is preferred for all dates.
- All observed time values will be presented using a 24-hour clock HH:MM:SS format (*e.g.,* 01:35:45 or 11:26). Seconds should only be reported if they were measured as part of the study.
- Time durations will be reported in mixed HHh MMm SSs notation (*e.g.*, 5h 32m, or 27h 52m 31s). The use of decimal notation to present time durations should be avoided (*e.g.* 0.083h = 5m) unless it is necessary to show the computation of time differences in a table, figure, or data listing, in which case both notations may be used to display the time duration.

10.2. Population Summary Conventions

- The biomarker concentration in serum determined at each sampling time point should be presented on the original scale for each subject participating in the study.
- All individual concentrations and efficacy parameters should be listed by visit together with summary statistics (n, arithmetic mean, median, standard deviation, coefficient of variation, minimum and maximum).
- All population summaries for continuous variables will include: N, mean, SD, median, minimum, and maximum.
- Population(s) represented on the tables or data listings will be clearly identified in the last title of the Table as "(<name of population>)" and will be identical in name to that identified in the protocol or SAP.

10.3. Biomarker Related Conventions

In the case where the absolute value in biomarker level is found below the lower limit of quantification, the absolute value will be arbitrarily set as 75% of the LOQ of the test to allow statistical treatment taking into consideration sample dilution.

10.4. Rounding

- Observed serum concentrations of Coll2-1 and CRP biomarkers will be respectively reported to five and three significant figures.
- P-values ≥0.001 will be reported to 3 decimal places; p-values less than 0.001 will be reported as "<0.001".

11. REFERENCES

[1] ICH Guidance Topic E9 "Statistical Principles for Clinical Trials"
[2] Pham et al. "Outcome Variables for Osteoarthritis Clinical Trials: The OMERACT-OARSI Set of Responder Criteria" The Journal of Rheumatology 2003; 30:7
[3] McCrum-Gardner E. "Which is the correct statistical test to use?". British journal of Oral and Maxillofacial Surgery 46 (2008) 38-41.

12. TABLES, FIGURES AND GRAPHS

12.1. Summary Tables and Figures

The following is a list of summary tables and figures that should be included in the report either in the text or in a dedicated chapter reporting all the tables, figures and graphs referred to but not included in the text. The numbering is given following the ICH structures of the chapter 14 of the "Structure and content of clinical study reports E3". All the tables and figures may be renumbered as appropriate during the compilation of the report. The listings and descriptive statistics already reported in the PK report are not included.

14.1 DEMOGRAPHIC DATA
14.1.1 Disposition of subjects and withdrawal
Table 14.1.1.1: Patients disposition
Table 14.1.1.2: Withdrawals reasons
Table 14.1.1.3: Median Follow-up
Table 14.1.1.4: Included patient by site
Table 14.1.1.5: Flow-chart - patients disposition
14.1.2 Protocol violations and deviations
Table 14.1.2.1: Study populations
Table 14.1.2.2: Protocol deviations
Table 14.1.2.3: Flow-chart - study populations

14.1.3 Demographic data
Table 14.1.3.1: Demographic data - FAS population
Table 14.1.3.2: Demographic data - Post-hoc tests if significant - FAS population
Table 14.1.3.3: Demographic data - PP population
Table 14.1.3.4: Demographic data - Post-hoc tests if significant - PP population
14.1.4 Prior and concomitant medication
Table 14.1.4.1: Prior medication by ATC name - Safety population
Table 14.1.4.2: Patients taking at least one concomitant treatment by ATC name
Table 14.1.4.3: Concomitant medication by ATC name - Safety population
Table 14.1.4.4: Concomitant medication by molecule - Safety population
14.1.5 Baseline conditions
14.1.5.1 Baseline medical history
Table 14.1.5.1.1: Significant medical/surgical history by SOP and PT - number of patients - Safety population
Table 14.1.5.1.2: Significant medical/surgical history by SOP and PT - Safety population
Table 14.1.5.1.3: Concomitant pathologies by SOC and PT - number of patients - Safety population
Table 14.1.5.1.4: Concomitant pathologies by SOC and PT - Safety population
Table 14.1.5.1.5: Treated concomitant pathologies by SOC and PT - number of patients - - Safety population
Table 14.1.5.1.6: Treated concomitant pathologies by SOC and PT - Safety population
14.1.5.2 Knee OA history, treatment history and pain assessment
Table 14.1.5.2.1: Knee OA history and pain assessment - FAS population
Table 14.1.5.2.2: Knee OA history and pain assessment - Post-hoc tests if significant - FAS population
Table 14.1.5.2.3: Knee OA history and pain assessment - PP population
Table 14.1.5.2.4: Knee OA history and pain assessment - Post-hoc tests if significant - PP population
Table 14.1.5.2.5: Knee OA treatment history - FAS population
Table 14.1.5.2.6: Knee OA treatment history - PP population
14.1.5.3 Biomarkers baseline data
Table 14.1.5.3.1: Biomarkers baseline data - FAS population
Table 14.1.5.3.2: Biomarkers baseline data - Post-hoc tests if significant - FAS population
Figure 14.1.5.3.3a: Biomarkers s-Coll2-1 concentration at baseline, per treatment group. nonparametric box-and-whiskers plots - FAS population
Figure 14.1.5.3.3b: Biomarkers ultrasensitive CRP concentration at baseline, per treatment group. nonparametric box-and-whiskers plots - FAS population
Table 14.1.5.3.4: Biomarkers baseline data - PP population
Table 14.1.5.3.5: Biomarkers baseline data - Post-hoc tests if significant - PP population
Figure 14.1.5.3.6a: Biomarkers s-Coll2-1 concentration at baseline, per treatment group. nonparametric box-and-whiskers plots - PP population
Figure 14.1.5.3.6b: Biomarkers ultrasensitive CRP concentration at baseline, per treatment group. nonparametric box-and-whiskers plots - PP population
:
14.2 EFFICACY DATA
14.2.1 Efficacy descriptive analysis
FAS population
Table 14.2.1: Efficacy descriptive Analysis: Biomarker sColl2-1 - FAS population
Table 14.2.2: Efficacy descriptive Analysis: Global assessment of disease activity (VAS) - FAS Population
Table 14.2.3: Efficacy descriptive Analysis: Ultrasensitive CRP - FAS Population
Table 14.2.4: Efficacy descriptive Analysis: Mean knee pain of the last 24 hours (VAS) - FAS Population
Table 14.2.5: Efficacy descriptive Analysis: KOOS scores (global KOOS score) - FAS Population
Table 14.2.6: Efficacy descriptive Analysis: KOOS scores (Pain sub-scores) - FAS Population

Table 14.2.7: Efficacy descriptive Analysis: KOOS scores (Symptoms sub-scores) - FAS Population
Table 14.2.8: Efficacy descriptive Analysis: KOOS scores (Function in daily living (ADL) sub-scores) - FAS Population
Table 14.2.9: Efficacy descriptive Analysis: KOOS scores (Function in sport and recreation (Sport/Rec) sub-scores) - FAS Population
Table 14.2.10: Efficacy descriptive Analysis: KOOS scores (knee related Quality of life (QOL) sub-scores) - FAS Population
Table 14.2.11: Efficacy descriptive Analysis: Pill count - FAS Population
Table 14.2.12: Efficacy descriptive Analysis: Curcumin level in serum (7 metabolites) - FAS Population
Table 14.2.13: Efficacy descriptive Analysis: Patient' satisfaction with treatment - FAS Population
Table 14.2.14: Efficacy descriptive Analysis: Use of oral rescue treatments for knee pain in the last month - FAS Population

PP population

Table 14.2.15: Efficacy descriptive Analysis: Biomarker sColl2-1 - PP population
Table 14.2.16: Efficacy descriptive Analysis: Global assessment of disease activity (VAS) - PP Population
Table 14.2.17: Efficacy descriptive Analysis: Ultrasensitive CRP - PP Population
Table 14.2.18: Efficacy descriptive Analysis: Mean knee pain of the last 24 hours (VAS) - PP Population
Table 14.2.19: Efficacy descriptive Analysis: KOOS scores (global KOOS score) - PP Population
Table 14.2.20: Efficacy descriptive Analysis: KOOS scores (Pain sub-scores) - PP Population
Table 14.2.21: Efficacy descriptive Analysis: KOOS scores (Symptoms sub-scores) - PP Population
Table 14.2.22: Efficacy descriptive Analysis: KOOS scores (Function in daily living (ADL) sub-scores) - PP Population
Table 14.2.23: Efficacy descriptive Analysis: KOOS scores (Function in sport and recreation (Sport/Rec) sub-scores) - PP Population
Table 14.2.24: Efficacy descriptive Analysis: KOOS scores (knee related Quality of life (QOL) sub-scores) - PP Population
Table 14.2.25: Efficacy descriptive Analysis: Pill count - PP Population
Table 14.2.26: Efficacy descriptive Analysis: Curcumin level in serum - PP Population
Table 14.2.27: Efficacy descriptive Analysis: Patient' satisfaction with treatment - PP Population
Table 14.2.28: Efficacy descriptive Analysis: Use of oral rescue treatments for knee pain in the last month - PP Population
Figure 14.2.29: Efficacy descriptive Analysis: Biomarker sColl2-1 (mean±SD) per treatment in function of time. Concentration-time courses - PP population
Figure 14.2.30: Efficacy descriptive Analysis: Global assessment of disease activity (VAS) (mean±SD) per treatment in function of time. Concentration-time courses - PP population
Figure 14.2.31: Efficacy descriptive Analysis: ultrasensitive CRP (mean±SD) per treatment in function of time. Concentration-time courses - PP population
Figure 14.2.32: Efficacy descriptive Analysis: Mean knee pain of the last 24 hours (VAS) (mean±SD) per treatment in function of time. Concentration-time courses - PP population
Figure 14.2.33: Efficacy descriptive Analysis: KOOS scores (global KOOS score) (mean±SD) per treatment in function of time. Concentration-time courses - PP population
Figure 14.2.34: Efficacy descriptive Analysis: KOOS scores (Pain sub-scores) (mean±SD) per treatment in function of time. Concentration-time courses - PP population
Figure 14.2.35: Efficacy descriptive Analysis: KOOS scores (Symptoms sub-scores) (mean±SD) per treatment in function of time. Concentration-time courses - PP population
Figure 14.2.36: Efficacy descriptive Analysis: KOOS scores (Function in daily living (ADL) sub-scores) (mean±SD) per treatment in function of time. Concentration-time courses - PP population
Figure 14.2.37: Efficacy descriptive Analysis: KOOS scores (Function in sport and recreation (Sport/Rec) sub-scores) (mean±SD) per treatment in function of time. Concentration-time courses - PP population
Figure 14.2.38: Efficacy descriptive Analysis: KOOS scores (knee related Quality of life (QOL) sub-scores) (mean±SD) per treatment in function of time. Concentration-time courses - PP population

Figure 14.2.39: Efficacy descriptive Analysis: KOOS Profile: mean KOOS score versus KOOS subscales, per timepoint. Treatment Group A - PP population
Figure 14.2.40: Efficacy descriptive Analysis: KOOS Profile: mean KOOS score versus KOOS subscales, per timepoint. Treatment Group B - PP population
Figure 14.2.41: Efficacy descriptive Analysis: KOOS Profile: mean KOOS score versus KOOS subscales, per timepoint. Treatment Group C - PP population
Figure 14.2.42: Efficacy descriptive Analysis: KOOS Profile: mean KOOS score versus KOOS subscales, per treatment. Baseline (T0) - PP population
Figure 14.2.43: Efficacy descriptive Analysis: KOOS Profile: mean KOOS score versus KOOS subscales, per treatment. T1 - PP population
Figure 14.2.44: Efficacy descriptive Analysis: KOOS Profile: mean KOOS score versus KOOS subscales, per treatment. T3 - PP population
Figure 14.2.45: Efficacy descriptive Analysis: KOOS Profile: mean KOOS score versus KOOS subscales, per treatment. T6 - PP population
Figure 14.2.46: Efficacy descriptive Analysis: curcumin level in serum (mean±SD) per treatment in function of time. Concentration-time courses - PP population

14.2.2 Primary Efficacy Data

FAS population

Table 14.2.2.1: Treatment responses: Biomarker sColl2-1, repeated measures ANCOVA model - FAS Population
Table 14.2.2.2: Treatment responses: Biomarker sColl2-1, post-hoc tests if significant - FAS Population
Table 14.2.2.3: Treatment responses: Global assessment of disease activity (VAS), repeated measures ANCOVA model - FAS Population
Table 14.2.2.4: Treatment responses: Global assessment of disease activity (VAS), post-hoc tests if significant - FAS Population

PP population

Table 14.2.2.5

Table 14.2.2.6: Treatment responses: Biomarker sColl2-1, post-hoc tests if significant - PP Population
Table 14.2.2.7: Treatment responses: Global assessment of disease activity (VAS), repeated measures ANCOVA model - PP Population
Table 14.2.2.8: Treatment responses: Global assessment of disease activity (VAS), post-hoc tests if significant - PP Population

Sensitivity analysis - PP population

Table 14.2.2.5: Treatment responses: Biomarker sColl2-1, repeated measures ANCOVA model - PP Population
Table 14.2.2.6: Treatment responses: Biomarker sColl2-1, post-hoc tests if significant - PP Population
Table 14.2.2.7: Treatment responses: Global assessment of disease activity (VAS), repeated measures ANCOVA model - PP Population
Table 14.2.2.8: Treatment responses: Global assessment of disease activity (VAS), post-hoc tests if significant - PP Population

14.2.3 Secondary Efficacy Data

FAS population

Table 14.2.3.1: Treatment responses: Biomarker sColl2-1, ANOVA model - FAS Population
Table 14.2.3.2: Treatment responses: Biomarker sColl2-1, post-hoc tests if significant - FAS Population
Table 14.2.3.3: Treatment responses: Global assessment of disease activity (VAS), repeated measures ANCOVA model - FAS Population
Table 14.2.3.4: Treatment responses: Global assessment of disease activity (VAS), post-hoc tests if significant - FAS Population
Table 14.2.3.5: Treatment responses: Ultrasensitive CRP, repeated measures ANCOVA model - FAS Population
Table 14.2.3.6: Treatment responses: Ultrasensitive CRP, post-hoc tests if significant - FAS Population
Table 14.2.3.7: Treatment responses: Mean knee pain of the last 24 hours (VAS), repeated measures ANCOVA model - FAS Population

Table 14.2.3.8: Treatment responses: Mean knee pain of the last 24 hours (VAS), post-hoc tests if significant - FAS Population
Table 14.2.3.9: Treatment responses: KOOS scores (global KOOS score), repeated measures ANCOVA model - FAS Population
Table 14.2.3.10: Treatment responses: KOOS scores (global KOOS score), post-hoc tests if significant - FAS Population
Table 14.2.3.11: Treatment responses: KOOS scores (Pain sub-scores), repeated measures ANCOVA model - FAS Population
Table 14.2.3.12: Treatment responses: KOOS scores (Pain sub-scores), post-hoc tests if significant - FAS Population
Table 14.2.3.13: Treatment responses: KOOS scores (Symptoms sub-scores), repeated measures ANCOVA model - FAS Population
Table 14.2.3.14: Treatment responses: KOOS scores (Symptoms sub-scores), post-hoc tests if significant - FAS Population
Table 14.2.3.15: Treatment responses: KOOS scores (Function in daily living (ADL) sub-scores), repeated measures ANCOVA model - FAS Population
Table 14.2.3.16: Treatment responses: KOOS scores (Function in daily living (ADL) sub-scores), post-hoc tests if significant - FAS Population
Table 14.2.3.17: Treatment responses: KOOS scores (Function in sport and recreation (Sport/Rec) sub-scores), repeated measures ANCOVA model - FAS Population
Table 14.2.3.18: Treatment responses: KOOS scores (Function in sport and recreation (Sport/Rec) sub-scores), post-hoc tests if significant - FAS Population
Table 14.2.3.19: Treatment responses: KOOS scores (knee related Quality of life (QOL) sub-scores), repeated measures ANCOVA model - FAS Population
Table 14.2.3.20: Treatment responses: KOOS scores (knee related Quality of life (QOL) sub-scores), post-hoc tests if significant - FAS Population
Table 14.2.3.21: Treatment responses: Pill count, Kruskall wallis test - FAS Population
Table 14.2.3.22: Treatment responses: Pill count, post-hoc tests if significant - FAS Population
Table 14.2.3.23: Treatment responses: Curcumin level in serum, repeated measures ANCOVA model - FAS Population
Table 14.2.3.24: Treatment responses: Curcumin level in serum, post-hoc tests if significant - FAS Population
Table 14.2.3.25: Treatment responses: Patient' satisfaction with treatment, Kruskall wallis test - FAS Population
Table 14.2.3.26: Treatment responses: Patient' satisfaction with treatment, post-hoc tests if significant - FAS Population
Table 14.2.3.27: Treatment responses: Use of oral rescue treatments for knee pain in the last month - yes/no, Kruskall wallis test - FAS Population
Table 14.2.3.28: Treatment responses: Use of oral rescue treatments for knee pain in the last month - yes/no, post-hoc tests if significant - FAS Population
Table 14.2.3.29: Treatment responses: Use of oral rescue treatments for knee pain in the last month - number of days, Kruskall wallis test - FAS Population
Table 14.2.3.30: Treatment responses: Use of oral rescue treatments for knee pain in the last month - number of days, post-hoc tests if significant - FAS Population

PP population

Table 14.2.3.1: Treatment responses: Biomarker sColl2-1, ANOVA model - PP Population
Table 14.2.3.2: Treatment responses: Biomarker sColl2-1, post-hoc tests if significant - PP Population
Table 14.2.3.3: Treatment responses: Global assessment of disease activity (VAS), repeated measures ANCOVA model - PP Population
Table 14.2.3.4: Treatment responses: Global assessment of disease activity (VAS), post-hoc tests if significant - PP Population
Table 14.2.3.5: Treatment responses: Ultrasensitive CRP, repeated measures ANCOVA model - PP Population
Table 14.2.3.6: Treatment responses: Ultrasensitive CRP, post-hoc tests if significant - PP Population

Table 14.2.3.7: Treatment responses: Mean knee pain of the last 24 hours (VAS), repeated measures ANCOVA model - PP Population
Table 14.2.3.8: Treatment responses: Mean knee pain of the last 24 hours (VAS), post-hoc tests if significant - PP Population
Table 14.2.3.9: Treatment responses: KOOS scores (global KOOS score), repeated measures ANCOVA model - PP Population
Table 14.2.3.10: Treatment responses: KOOS scores (global KOOS score), post-hoc tests if significant - PP Population
Table 14.2.3.11: Treatment responses: KOOS scores (Pain sub-scores), repeated measures ANCOVA model - PP Population
Table 14.2.3.12: Treatment responses: KOOS scores (Pain sub-scores), post-hoc tests if significant - PP Population
Table 14.2.3.13: Treatment responses: KOOS scores (Symptoms sub-scores), repeated measures ANCOVA model - PP Population
Table 14.2.3.14: Treatment responses: KOOS scores (Symptoms sub-scores), post-hoc tests if significant - PP Population
Table 14.2.3.15: Treatment responses: KOOS scores (Function in daily living (ADL) sub-scores), repeated measures ANCOVA model - PP Population
Table 14.2.3.16: Treatment responses: KOOS scores (Function in daily living (ADL) sub-scores), post-hoc tests if significant - PP Population
Table 14.2.3.17: Treatment responses: KOOS scores (Function in sport and recreation (Sport/Rec) sub-scores), repeated measures ANCOVA model - PP Population
Table 14.2.3.18: Treatment responses: KOOS scores (Function in sport and recreation (Sport/Rec) sub-scores), post-hoc tests if significant - PP Population
Table 14.2.3.19: Treatment responses: KOOS scores (knee related Quality of life (QOL) sub-scores), repeated measures ANCOVA model - PP Population
Table 14.2.3.20: Treatment responses: KOOS scores (knee related Quality of life (QOL) sub-scores), post-hoc tests if significant - PP Population
Table 14.2.3.21: Treatment responses: Pill count, Kruskall wallis test - PP Population
Table 14.2.3.22: Treatment responses: Pill count, post-hoc tests if significant - PP Population
Table 14.2.3.23: Treatment responses: Curcumin level in serum, repeated measures ANCOVA model - PP Population
Table 14.2.3.24: Treatment responses: Curcumin level in serum, post-hoc tests if significant - PP Population
Table 14.2.3.25: Treatment responses: Patient' satisfaction with treatment, Kruskall wallis test - PP Population
Table 14.2.3.26: Treatment responses: Patient' satisfaction with treatment, post-hoc tests if significant - PP Population
Table 14.2.3.27: Treatment responses: Use of oral rescue treatments for knee pain in the last month - yes/no, Kruskall wallis test - PP Population
Table 14.2.3.28: Treatment responses: Use of oral rescue treatments for knee pain in the last month - yes/no, post-hoc tests if significant - PP Population
Table 14.2.3.29: Treatment responses: Use of oral rescue treatments for knee pain in the last month - number of days, Kruskall wallis test - PP Population
Table 14.2.3.30: Treatment responses: Use of oral rescue treatments for knee pain in the last month - number of days, post-hoc tests if significant - PP Population

12.2. Data Listings

The following is a list of data-listing that should be included as appendix to the report. The numbering is given following the ICH structures of the chapter 16 of the "Structure and content of clinical study reports E3". All the tables may be renumbered as appropriate during the compilation of the report.

16.2 PATIENT DATA LISTINGS

16.2.1 Discontinued patients

Listing 16.2.1.1: Discontinued patients

16.2.2 Protocol deviations

Listing 16.2.2.1: Protocol deviations

16.2.3 Patients excluded from the efficacy analysis

Listing 16.2.3.1: Patients excluded from the efficacy analysis

16.2.4 Demographic data

Listing 16.2.4.1: Demographic data
Listing 16.2.4.2: Prior and Concomitant Medications
Listing 16.2.4.3: Medical history and concomitant pathologies
Listing 16.2.4.4: Knee OA history
Listing 16.2.4.5: Knee OA pain assessment
Listing 16.2.4.6: Knee OA treatment history
Listing 16.2.4.7: Biomarkers Baseline Data

16.2.5 Compliance and/or drug concentration data (if available)

Listing 16.2.5.1: Pill count and curcumin blood level

16.2.6 Individual efficacy response data

Listing 16.2.6.1: Coll2-1
Listing 16.2.6.2: Global assessment of disease activity (VAS)
Listing 16.2.6.3: Ultrasensitive CRP
Listing 16.2.6.4: Mean knee pain over the last 24 hours (VAS)
Listing 16.2.6.5: Global KOOS and its subscales
Listing 16.2.6.6: Rescue tretments for knee pain in the last month
Listing 16.2.6.7: Patient' satisfaction with treatment

16.2.7 Adverse event listings (each patient)

Listing 16.2.7: Listing of AE - safety population

16.2.8. Listing of individual laboratory measurements by patient, when required by regulatory authorities

Listing 16.2.8.1: Laboratory values for subjects with AE linked to the product
